# Trajectories of sleep health during the perinatal period: a systematic review and meta-analysis

**DOI:** 10.1093/sleep/zsaf095

**Published:** 2025-04-28

**Authors:** Man Wang, Jialu Qian, Youngmin Cho, Zhiting Guo, Xiaoyan Yu, Junxin Li

**Affiliations:** Department of Nursing, Zhejiang University School of Medicine, Hangzhou, China; Center for Equity in Aging, Johns Hopkins University School of Nursing, Baltimore, MD, USA; School of Nursing, Nanjing University of Chinese Medicine, Nanjing, China; Center for Equity in Aging, Johns Hopkins University School of Nursing, Baltimore, MD, USA; Department of Nursing, Zhejiang University School of Medicine, Hangzhou, China; Center for Equity in Aging, Johns Hopkins University School of Nursing, Baltimore, MD, USA; Department of Obstetrics and Gynecology, Women’s Hospital School of Medicine Zhejiang University, Hangzhou, China; Center for Equity in Aging, Johns Hopkins University School of Nursing, Baltimore, MD, USA

**Keywords:** sleep health, trajectory, heterogeneity, perinatal period, systematic review, meta-analysis

## Abstract

**Study Objectives:**

This review aims to summarize trajectories of sleep quality, duration, efficiency, timing, and insomnia symptoms from pregnancy to 1 year postpartum, with a specific focus on identifying the number, proportion, shape, associated factors, and outcomes of these trajectories.

**Methods:**

We conducted a systematic search across eight databases from inception to August 13, 2024. Longitudinal studies that recruited 100 or more pregnant or postpartum women with at least three sleep assessments during pregnancy and 1 year postpartum, and modeled independent sleep health trajectories using trajectory analysis methods were included. Meta-analyses were performed to assess the pooled prevalence of nonoptimal sleep health trajectories. The prevalence was compared across geographical regions by subgroup meta-analysis. Group-based trajectory model (GBTM) was used to reidentify clusters of sleep health trajectories if available.

**Results:**

Five studies modeled a single trajectory, and the other 12 studies identified two to four distinct trajectories. The pooled prevalence of nonoptimal sleep quality and duration trajectories was 36% and 22%, respectively. The mean prevalence of the nonoptimal sleep efficiency trajectory was 15%, while the prevalence of delayed bedtime, late wake-up time, and clinical insomnia trajectories was reported as 51%, 17%, and 13%, respectively. Nonoptimal sleep trajectories were associated with higher risks of adverse maternal and infant outcomes. Low socioeconomic status, high pre-pregnancy body mass index, poor baseline sleep quality and self-reported health, and high initial levels of fatigue, anxiety, and depressive symptoms were key factors associated with these trajectories. Additionally, GBTM identified three trajectory groups of perinatal sleep quality: consistently good (38.9%), increasingly poor (37.6%), and decreasingly poor (23.5%).

**Conclusions:**

Perinatal sleep health trajectories demonstrate significant heterogeneity, with a notable proportion of women following high-risk trajectories. Further research should focus on identifying key risk factors for sleep health trajectories early in the perinatal period and developing targeted public health strategies and interventions to address these factors.

**Registration:**

PROSPERO registration: CRD42023407670.

## Graphical Abstract

**Graphical Abstract:**
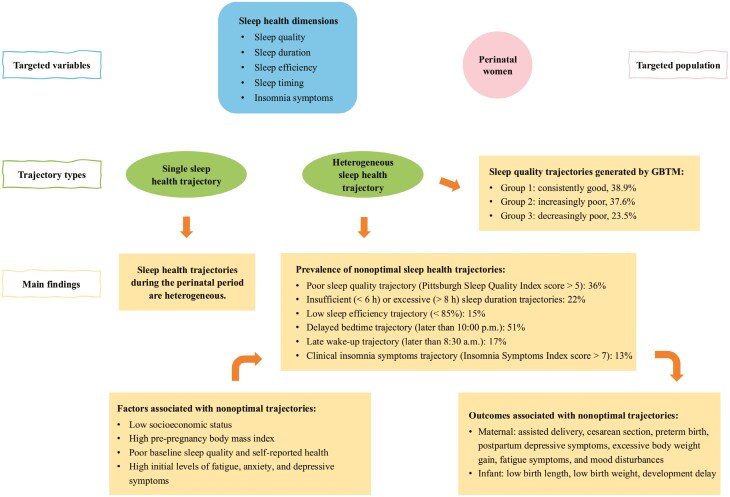

Sleep health is vital for health and well-being in perinatal women [1, 2]. Good sleep health plays a critical role in reducing the risk of adverse maternal and fetal outcomes [3]. Good sleep health requires satisfactory quality, adequate duration, high efficiency, appropriate timing, and sustained alertness during waking hours [4]. However, perinatal women often complain about poor sleep health such as poor sleep quality, insufficient sleep duration, low sleep efficiency, abnormal sleep timing and daytime sleepiness [5]. Two recent meta-analyses pointed out that 45.7% of pregnant women experience poor sleep quality, and 38.2% report insomnia symptoms such as delayed bedtime, low sleep efficiency, and early morning awakening [6, 7]. Sleep complaints often persist into the postpartum period, sometimes lasting nearly 1 year after childbirth [8, 9]. Multiple studies have demonstrated that poor sleep health increases the risks of adverse maternal and infant outcomes, including gestational hypertension, gestational diabetes mellitus, preterm birth, unplanned cesarean section (C-section), large for gestational age, and postpartum depression (PPD) [3, 10]. These findings emphasize the critical role of maintaining good sleep health in promoting the well-being of both mothers and their infants.

Sleep health undergoes significant changes during the perinatal period, which spans pregnancy through the first 12 months postpartum [11]. These changes are influenced by different challenges related to specific phases of pregnancy and postpartum, such as hormonal fluctuations, frequent urination, fetal movements, and discomfortable sleep positions due to the growing belly or breastfeeding [12, 13]. Furthermore, women may experience different sleep challenges due to the presence of individual differences (e.g. socioeconomic status, family structure, and home environment), which contribute to the heterogeneity of sleep health trajectories. Specifically, women with low socioeconomic status were more likely to experience persistent severe sleep problems, while others maintained relatively good sleep health [14–16]. Additionally, existing literature have confirmed that sleep quality [14, 15, 17], sleep duration [18, 19], sleep efficiency [20], sleep timing [21], and insomnia symptoms [22] exhibit heterogeneous trajectories. Notably, only specific trajectories of sleep health dimensions were linked to increased risks of preterm birth [18], PPD symptoms [15] in mothers, and overall developmental delay in infants [18].

Most existing systematic reviews primarily summarize sleep health status based on cross-sectional data [23–25], which limits a holistic understanding of longitudinal sleep change patterns and their heterogeneity during the perinatal period. Additionally, these reviews often focus on individual dimensions of sleep health (e.g. sleep quality or sleep duration) without integrating multiple dimensions to capture its full complexity. In light of significant changes in multiple dimensions of sleep health during the perinatal period, it is valuable to systematically review all available studies that investigated sleep health trajectories among perinatal women. To the best of our knowledge, the findings of these studies have not yet been systematically synthesized. Therefore, this systematic review and meta-analysis summarize studies that have examined trajectories of multiple sleep health dimensions from pregnancy to the first year postpartum, including sleep quality, duration, efficiency, timing, and insomnia symptoms. Our aims were to understand the number, shape, proportion, associated factors, and outcomes of sleep health trajectories, informing public health policies for identifying high-risk trajectory groups.

## Methods

This systematic review was conducted in accordance with the Preferred reporting items for systematic reviews and meta-analyses (PRISMA) guidelines [26]. The study protocol was registered with the International Prospective Register of Systematic Reviews (PROSPERO; registration number: CRD42023407670).

### Search strategy

A systematic literature search was conducted from the inception of each database to August 13, 2024, without any restrictions on the start date, to ensure a comprehensive review of all relevant studies on this topic. The databases searched included six English databases (PubMed, Embase, CINAHL, Scopus, PsycINFO, and Web of Science) and two Chinese databases (CNKI and Wanfang Data). Search terms related to (1) perinatal period, (2) sleep, and (3) trajectory were modified based on the target database, and a language filter was applied to limit the results to articles written in English or Chinese (Table S1). We additionally reviewed the reference lists of included studies to identify other potentially eligible studies.

### Eligibility criteria

Studies were included if they: (1) were longitudinal quantitative studies, (2) recruited pregnant women or postpartum women (within 1 year after childbirth), (3) had at least three sleep assessments during pregnancy and within the first year postpartum, and (4) employed trajectory analysis methods to describe the overall sleep change pattern over time (single sleep trajectory) or identify distinct subgroups following a similar pattern of sleep change over time (heterogeneous sleep trajectories), for example, latent growth curve modeling, group-based trajectory modeling (GBTM), growth mixture modeling (GMM), and latent class growth analysis. Studies were excluded if they: (1) were not published in English or Chinese; (2) had a sample size of fewer than 100, which may compromise the performance and convergence of trajectory modeling [27]; (3) jointly modeled trajectories of multiple sleep parameters or sleep parameters with other traits (e.g. depressive symptoms), but not independent sleep trajectories.

### Quality assessment

The methodological quality was independently evaluated by two authors (MW and JQ) using the Newcastle-Ottawa quality assessment scale (NOS) for cohort studies, which employs a star rating system based on three domains: selection (four items), comparability (one item), and outcome (three items) [28]. Studies were categorized as good, fair, and poor quality based on scores of the three domains (Table S3 for details of the rating system). Studies of poor quality as determined by the NOS were not excluded, but the reliability of the results will be considered carefully. To assess the reporting quality and transparency of studies identifying heterogeneous sleep trajectories, two authors (MW and JQ) independently evaluated them using the Guidelines for Reporting on Latent Trajectory Studies (GRoLTS) [29]. The checklist consists of 21 items that examine whether essential aspects such as metric of time, software used, handling of missing data, model comparisons, and visualization of final trajectories are adequately reported. Each item was scored as 0 (not reported) or 1 (reported), with the total GRoLTS score ranging from 0 to 21. While the GRoLTS criteria do not define specific cutoff points, a higher score reflects better reporting quality. Any disagreements in the quality assessment were resolved by consensus discussion, with a senior author (JL) adjudicating when necessary.

### Screening and data extraction

Studies were selected using Covidence (www.covidence.org). Titles, abstracts, and the full texts of relevant studies were initially screened by one author (MW) and then double-checked by the second author (JQ). Data extracted from each included study by the two authors (MW and JQ) encompassed: author, year of publication, study location, sample characteristics (e.g. sample size and age together with mean or median), sleep dimensions (e.g. measures and time points), trajectory analysis methods and results, as well as health outcomes (e.g. maternal pregnancy outcomes and infant birth outcomes) associated with sleep trajectories. If any studies had incomplete or unclear information, the authors were contacted to obtain additional details to ensure the completeness and accuracy of the data.

### Data synthesis

A narrative synthesis was performed based on the types of sleep trajectory (single or heterogeneous) and further subdivided based on sleep dimensions, such as sleep quality, sleep duration, sleep efficiency, and others. We also summarized associated factors and health-related outcomes. We conducted random-effects meta-analyses to estimate the pooled prevalence of participants experiencing nonoptimal sleep health trajectories, defined by consistent deviations (either below or above) from established optimal sleep measures in prior research. Specifically, we defined nonoptimal sleep trajectories as follows: (1) nonoptimal sleep quality trajectory: Pittsburgh Sleep Quality Index (PSQI) scores persistently above five at all time points [30]; (2) nonoptimal sleep duration trajectory: self-reported sleep duration consistently less than 6 hours or more than 8 hours at all time points [31]; (3) nonoptimal sleep efficiency trajectory: sleep efficiency below 85% at all time points [32]; (4) nonoptimal bedtime trajectory: a delayed bedtime beyond 10:30 pm at all time points [21]; (5) nonoptimal wake-up time trajectory: waking up later than 08:00 am at all time points [21]; and (6) nonoptimal insomnia symptoms trajectory: Insomnia severity index (ISI) scores greater than seven at all time points [33]. Meta-analyses of prevalence often yield high I^2^ statistics, we therefore also reported prediction intervals to measure the heterogeneity when three or more studies were included in one meta-analysis [34, 35]. Subgroup meta-analyses were performed to examine the differences in the prevalence of nonoptimal sleep health trajectories across diverse geographical regions (e.g. Asia, North America, and Europe). Publication bias was assessed using funnel plots where 10 or more studies were included in one meta-analysis [36].

To assess the robustness of our meta-analysis results, we planned sensitivity analyses by excluding low-quality studies (as assessed using the NOS) and outlier studies, if present. A study was classified as an outlier if its confidence interval (CI) did not overlap with the CI of the pooled effect [37]. Such studies were considered to have a high risk of bias or represent extreme values that could potentially influence the pooled prevalence. We additionally assessed inter-rater reliability using Cohen’s kappa for the NOS categorical ratings and interclass correlation coefficient (ICC) for the GRoLTS continuous scores. Cohen’s kappa values were interpreted as follows: <0.20 (poor), 0.21 to 0.40 (fair), 0.41 to 0.60 (moderate), 0.61 to 0.80 (good), and 0.81 to 1.00 (excellent) [38]. ICC values were classified as follows: <0.50 (poor), 0.50 to 0.75 (moderate), 0.75 to 0.90 (good), and >0.90 (excellent) [39].

To better synthesize the results of heterogeneous sleep trajectories, we extracted time points and corresponding sleep data from those studies to create a new dataset for each identified trajectory (Table S2). Sleep data prior to pregnancy were excluded from the GBTM analysis. Data during pregnancy were categorized into three groups based on gestational age (GA) as reported by the included studies: first trimester (<14 weeks GA), second trimester (14 to 27 weeks GA), and third trimester (>28 weeks GA). Postpartum data after pregnancy were retained as classified in the original studies. GBTM was employed to reidentify clusters of sleep health trajectories when the sleep data included 100 or more observations, as this threshold is recommended to ensure the robustness of this method [40]. Bayesian information criterion, Akaike information criterion (AIC), entropy, and average posterior probability (AvePP) were selected to determine the optimal model fit with the sleep data [29]. All analyses were performed using SAS 9.4 and STATA 18.0.

## Results

### Study selection and quality

**Figure 1.**
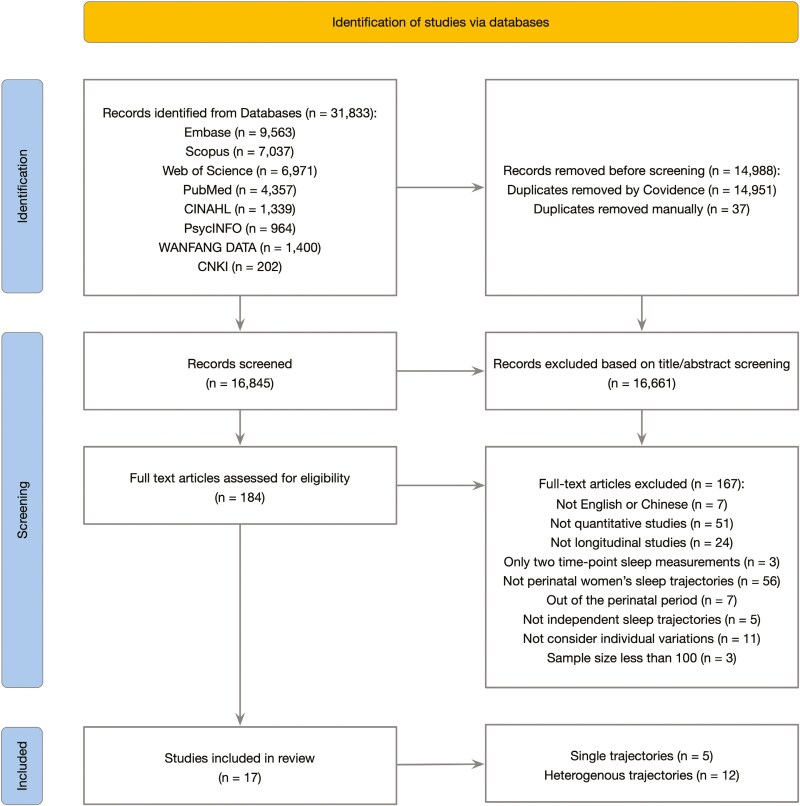
Flow diagram for study selection.

The study selection process is illustrated in Figure 1. The database search yielded a total of 31 833 initial records. After duplicate removal and title or abstract screening, 184 records were eligible for full-text screening. The three main reasons for exclusion were not perinatal women’s sleep trajectory (*n* = 56), not quantitative study (*n* = 51), and not longitudinal study (*n* = 24). Finally, 17 studies were included in this review with a total of 5033 perinatal women [8, 14–22, 41–47]. According to the NOS, 14 out of 17 studies were rated as good quality [8, 15–21, 41–45, 47], one study was rated as fair quality because the outcome was already present at the start of this cohort study [14], and two studies were classified as poor quality due to the use of inappropriate statistical methods and a low follow-up rate [22, 46] (Table S3). Based on GRoLTS criteria, among the 12 studies investigating heterogeneous sleep trajectories [14–22, 41–43], the highest score was 11 out of a possible 21, with a median score of seven (Table S4). The four most frequently reported methodological details in the included studies were: the metric of time used (Item 1), the software used for analysis (Item 5), the model selection tool employed (Item 10), and the final plot of trajectories (Item 14a). In contrast, several key aspects were not reported in any of the included studies, including alternative specifications of within-class heterogeneity (F1) and between-class differences (F2), inclusion and handling of covariates (H), the number of random start values and final iterations (I), the estimated mean trajectories for each model (N2), the estimated means of the final model and the observed trajectories (N3), and syntax files used for the analysis (P). The Cohen’s kappa for NOS was 0.81, and the ICC for GRoLTS was 0.84, both indicating good to excellent inter-rater reliability between the authors.

### Study characteristics of the included studies

Tables 1 and 2 present an overview of the characteristics of the 17 included studies for single sleep trajectory (*n* = 5) [8, 44–47] and heterogeneous sleep trajectories (*n* = 12) [14–22, 41–43], respectively. They published between 2015 and 2024, covering a wide range of countries, including Asia (*n* = 10) [8, 14, 16–18, 21, 41–43, 45], North America (*n* = 4) [15, 20, 22, 47], Europe (*n* = 2) [19, 46], and Australia (*n* = 1) [44]. The sample size ranged from 120 to 1192. All included studies reported the mean age of participants, which ranged from 22.56 [21] to 33.6 years [18]. Eleven studies provided data on annual or monthly household income [8, 15, 16, 18, 21, 22, 41, 43–45, 47], indicating that a relatively high proportion of participants had incomes above the average level in their respective regions. Almost all studies reported that the majority of participants had a college degree or higher [8, 14–18, 20–22, 41–46]. Employment status was reported in six studies, which indicated that a large proportion of participants were employed [8, 14, 17, 18, 42, 44]. In terms of parity, four studies only recruited primiparas [17, 41, 44, 45], while ten studies included both primiparas and multiparas [8, 14, 15, 18–22, 42, 46]. Three studies did not specify parity distribution [16, 43, 47]. Regarding pregnancy characteristics, 10 studies stated that their samples consisted of singleton pregnancies [8, 14, 17, 18, 21, 41, 43–45, 47], while the remaining studies did not report this information. Additionally, across the included studies, the majority of participants were healthy and married. All the studies are longitudinal, with 12 of them being cohort studies [14–22, 42, 43, 46]. Out of the 12 studies on heterogenous sleep trajectories, 10 employed GBTM [14–20, 22, 42, 43], 1 adopted GMM [41], and 1 used latent class growth analysis [21]. Five single sleep trajectory studies utilized latent growth curve modeling (*n* = 1) [46], mixed-effects model (*n* = 3) [44, 45, 47], or growth multilevel model (*n* = 1) [8]. All the studies employed subjective measures, except for Horwitz et al., 2023 [8], which employed a combination of subjective sleep diary and objective actigraphy. Assessment of time points ranged from three (*n* = 9) [16–21, 41, 45, 46] to seven (*n* = 1) [47]. While 11 studies included both pregnancy and postpartum time points [8, 14–16, 22, 41–45, 47], eight of them only included a single time point during either pregnancy [8, 16, 41–43, 45, 47] or postpartum [22].

**Table 1:** Overview of the Included Studies for Heterogeneous Sleep Trajectories (*n* = 12)

Study	Country	*N*	Age	Sleep dimensions and measures	Assessments	Methods	Trajectory groups
Bao et al., 2022 [16]	Wenzhou, Mainland China	412	28.5 (4.09)	Sleep quality (PSQI)	36 GA 1 week 6 weeks	GBTM	Four trajectories: Stable-good (48.1%) Worsening (18.4%) Improving (17.5%) Stable-poor (16.0%)
Li et al., 2023 [18]	Taiwan	1192	33.6 (3.88)	Sleep duration (Self-report question)	Early pregnancy Mid pregnancy Late pregnancy	GBTM	Four trajectories: Extremely long decreasing (2.9%) Stably adequate (43.5%) Stably short (51.1%) Short decreasing (2.4%)
Li et al., 2024 [21]	Tianjin, Mainland China	232	22.56 (3.69)	Sleep duration (PSQI) Sleep efficiency (PSQI) Bedtime (PSQI) Wake-up time (PSQI)	7–14 GA 20–27 GA 30–37 GA	LCGA	Three trajectories of sleep duration: Short sleep duration (5.2%) Adequate sleep duration (78.0%) Excessive sleep duration (16.8%) Two trajectories of sleep efficiency: High sleep efficiency (88.4%) Decreasing sleep efficiency (11.6%) Two trajectories of bedtime: Delaying bedtime (50.9%) Early bedtime (49.1%) Two trajectories of wake-up time: Early wake-up time (82.8%) Late wake-up time (17.2%)
Lin-Lewry et al., 2023 [14]	Taiwan	190	32.3 (4.1)	Sleep quality (PSQI)	28 GA 36 GA 1 week 1 month 3 months	GBTM	Three trajectories: Stable good (18.4%) Increasing poor (48.9%) Stable poor (32.6%)
Lyu et al., 2020 [17]	Shanghai, Mainland China	403	29.83 (3.41)	Sleep quality (PSQI)	10–17 GA 18–21 GA 28–32 GA	GBTM	Two trajectories: Good-poor (76.42%) Constant poor (23.57%)
Plancoulaine et al., 2017 [19]	Saint-Etienne, France	200	30 (18–43)	Sleep duration (Self-report questionnaire)	Pre-pregnancy First trimester Second trimester Third trimester	GBTM	Three trajectories: Short-decreasing (10.5%) Medium-decreasing (57.6%) Long-increasing (31.9%)
Sedov et al., 2020 [22]	Calgary, Canada	142	31.35 (4.51)	Insomnia symptoms (ISI)	<20 GA 20–29 GA 30–39 GA 6 weeks	GBTM	Three trajectories: No insomnia (42.25%) Subclinical insomnia (44.33%) Clinical insomnia (13.43%)
Tzeng et al., 2015 [39]	Taiwan	139	33.6 (3.8)	Sleep quality (PSQI)	36 GA 1 day 1 week 4 weeks 6 months	GBTM	Three trajectories: Stable poor sleep (36.0%) Progressively worse sleep (48.2%) Persistently poor sleep (15.8%)
Tomfohr et al., 2015 [15]	Calgary, Canada	293	30.9 (3.79)	Sleep quality (PSQI)	<22 GA 32 GA 3 months 6 months	GBTM	Four trajectories: Stable-low sleep complaints (21.51%) Increasing-mild sleep complaints (59.45%) Increasing-high sleep complaints (12.32%) Stable-high sleep complaints (6.71%)
Wang et al., 2018 [43]	Shanghai, Mainland China	262	29.39 (3.21)	Sleep quality (PSQI)	36–38 GA 42 days 3 months 6 months 9 months 12 months 18 months 24 months 36 months	GBTM	Three trajectories: Stable-low (29.4%) Decreasing-mild (56.5%) Stable-high (14.1%)
Whitaker et al., 2021 [20]	Pittsburgh and Iowa, United States	120	31.2 (4.8)	Sleep quality (PSQI) Sleep duration (PSQI) Sleep efficiency (PSQI)	8–13 GA 20–22 GA 32–34 GA	GBTM	Three trajectories of sleep quality: Poor quality (15.1%) Worsening quality (23.5%) Good quality (61.5%) Two trajectories of sleep duration: Adequate sleep duration (79.3%) Short sleep duration (20.7%) Two trajectories of sleep efficiency: Low sleep efficiency (17.5%) High sleep efficiency (82.5%)
Zhang et al., 2023 [41]	Wenzhou, Mainland China	140	27.01 (4.38)	Sleep quality (PSQI)	28–40 GA 24 hours 1 week	GMM	Three trajectories: High sleep quality (27.14%) Low sleep quality (22.14%) Decreasing sleep quality (50.71%)

PSQI, Pittsburgh sleep quality index; ISI, Insomnia severity index; GBTM, Group-based trajectory modeling; GMM, Growth mixture modeling; LCGA, Latent class growth analysis.

**Table 2.** Overview of the Included Studies for Single Sleep Trajectories (*n* = 5)

Study	Country	*N*	Age	Sleep dimensions and measures	Assessments	Methods	Trajectory trends
Nagi et al., 2024 [45]	Hongkong	231	32.1 (3.9)	Sleep quality (PSQI)	12–20 GA 6 weeks 6 months	Generalized linear mixed model	Sleep quality declined from pregnancy to 6 weeks postpartum, then improved from 6 weeks postpartum to 6 months postpartum.
Paul et al., 2019 [47]	Ohio and Colorado, United States	151	29 (5.2)	Sleep duration (SD)	32–36 GA 1 week 2 weeks 1 month 2 months 3 months 6 months	Generalized linear mixed effects model	Sleep duration slightly increased from third trimester to 6 months postpartum (slope = 0.02^*^).
van der Zwan et al., 2017 [46]	Turku, Finland	531	31.6 (4.4)	Sleep duration (BNSQ)	14 GA 24 GA 34 GA	Latent growth curve model	Sleep duration decreased from 14 weeks to 34 weeks of pregnancy (slope = −0.07^*^).
Verma et al., 2024 [44]	Melbourne, Australia	163	33.35 (3.42)	Bedtime (CSD) Rise-time (CSD) Chronotype (rMEQ)	30 GA 36 GA 1.5 months 3 months 6 months 12 months 24 months	Mixed-effects model	Bedtime and rise-time were delayed before childbirth and became earlier postpartum. Chronotype shifted to an evening-oriented type before childbirth and towards a more morning-oriented type postpartum.
Horwitz et al., 2023 [8]	Beer-Sheva, Israel	232	29.77 (3.14)	Sleep duration (SD) Sleep efficiency (SD) Night waking (SD) Sleep duration (Act) Sleep efficiency (Act) Night waking (Act) Longest sleep period (Act) Insomnia symptoms (ISI)	Third trimester 4 months 8 months 12 months	Growth multilevel model (two-slope piecewise model)	Sleep duration (SD): A linear decline before 4 months (slope = −7.50^*^) and a U-shaped trend after that (slope^2^ = 8.40^*^). Sleep efficiency (SD): A linear decline (slope = −5.13^**^) before 4 months and a linear increase after that (slope = 1.63^*^). Night waking (SD): A linear increase (slope = 0.61^**^) before 4 months and a linear decline after that (slope = −0.39^*^). Sleep duration (Act): Stable before 4 months and a U-shaped trend after that (slope^2^ = 10.22^*^). Sleep efficiency (Act): A linear decline (slope = −3.44^**^) before 4 months and stable after that. Night waking (Act): Stable. Longest sleep period (Act): A linear decline (slope = −19.99^**^) before 4 months and a quadratic increase after that (slope^2^ = 10.39^*^). Insomnia symptoms (ISI): A linear decline (slope = −0.54^**^) before 4 months and a reverse U-shaped trend after that (slope^2^ = 0.81^*^).

PSQI, Pittsburgh sleep quality index; SD, Sleep diary; BNSQ, Basic Nordic Sleep Questionnaire; ACT, Actigraphy; ISI, Insomnia severity index; CSD, Consensus sleep diary; rMEQ, the reduced Morningness–Eveningness Questionnaire. ^*^ <.05, ^**^ <.005, <.001. NR, Not report.

### Single sleep trajectory

#### Sleep quality, sleep duration, and sleep efficiency.

A significant decline in sleep quality was observed from pregnancy (PSQI = 5.52) to 6 weeks postpartum (PSQI = 7.05), followed by a recovery from 6 weeks to 6 months postpartum (PSQI = 5.18) [45]. Subjective sleep duration presented a decline from early to late pregnancy (slope = −0.07, *p* < .05) [46]. Findings on the trajectory of subjective sleep duration from late pregnancy to 6 months postpartum were inconsistent, with one study reporting a linear increase [47], while another observed a decrease [8]. Horwitz et al., 2023 found that both subjective and objective sleep duration showed a similar U-shaped trend from 4 to 12 months postpartum (quadratic slopes = 8.40 and 10.22, respectively, with *p* < .05), with a turning point at 8 months postpartum [8]. However, the change patterns of sleep duration from late pregnancy to 4 months postpartum differed between objective and subjective measures, with subjective reports indicating a decline, while objective measurements remained stable [8]. For objective and subjective sleep efficiency, there was a linear decline from late pregnancy to 4 months postpartum (slopes = −5.13 and −3.44, respectively, with *p* < .05). However, from 4 to 12 months postpartum, the change patterns diverged: subjective sleep efficiency gradually increased, while objective sleep efficiency remained stable.

#### Other sleep dimensions.

Verma et al., 2024 identified a quadratic trend in sleep timing and chronotype from late pregnancy to the first year postpartum [44]. Both bedtime and wake-up time were delayed by 8 and 20 minutes, respectively, from 30 to 36 weeks of pregnancy. They then shifted progressively earlier during the first year postpartum, and even earlier than the initial levels. Similarly, their chronotype shifted to an evening-oriented type before childbirth, and then gradually shifted towards a more morning-oriented type postpartum. Insomnia symptoms significantly increased from the third trimester of pregnancy to 4 months postpartum (slope = 0.54, *p* < .05), then showed a U-shaped trend from 4 to 12 months postpartum (quadratic slope = 0.81, *p* < .05), with a turning point at 8 months [8]. Subjective night-wakings increased from the third trimester of pregnancy to 4 months postpartum (slope = 0.61, *p* < .05), then decreased from 4 to 12 months (slope = -0.39, *p* < .05). However, no significant change was observed for objective night-wakings.

### Heterogeneous sleep trajectories

#### Sleep quality.

Eight studies modeled heterogenous sleep quality trajectories [14–17, 20, 41–43]. All of them employed PSQI and identified two [17], three [14, 20, 41–43], or four [15, 16] heterogenous sleep quality trajectories, respectively (Figure S1). These trajectories differed in two characteristics: stability (decreasing, stable, or increasing) and severity (low, moderate, or high). Specifically, six studies identified a single trajectory characterized by stable and low PSQI scores [14–17, 20, 43], consistently below or fluctuating around its cutoff point of five [30], indicating good sleep quality (PSQI ≤ 5). The proportion of participants in this trajectory group ranged from 21.51% [15] to 76.4% [17]. Five studies identified a stable poor sleep quality trajectory [16, 17, 20, 41, 43], characterized by PSQI scores above seven. This trajectory group had the lowest proportion of participants, ranging from 14.1% [43] to 23.57% [17]. Another small but significant trajectory group was identified by three studies [14, 16, 42], characterized by improved sleep quality during the puerperium. Another three studies identified a trajectory characterized by worsening sleep quality from late pregnancy to 1 week postpartum [14, 41, 42], accounting for approximately half of the participants.

The pooled prevalence of perinatal women following a nonoptimal sleep quality trajectory [14–17, 20, 43], with PSQI scores persistently above five, was 36% (95% CI: 20% to 51%; I^2^ = 98.3%; 95% PI: 0% to 97.5%; Figure S5-A). Two studies were classified as outliers and were removed from the meta-analysis [14, 43], which reduced the prevalence to 33% (95% CI: 17% to 49%; I^2^ = 97.4%; 95% PI: 0% to 100%; Figure S5-B). The small sample sizes of these studies were likely the reason for their classification as outliers. However, the reduction was <3%, indicating that the analysis remained robust after excluding outlier studies. No studies were rated as low quality, which reflects the reliability of our results. Subgroup meta-analysis by geographical region showed that the prevalence of nonoptimal sleep quality trajectory in Asia (39%) [14, 16, 17, 43] was higher than in North America (28%) [14, 43] (Figure S5-C).

**Figure 2.**
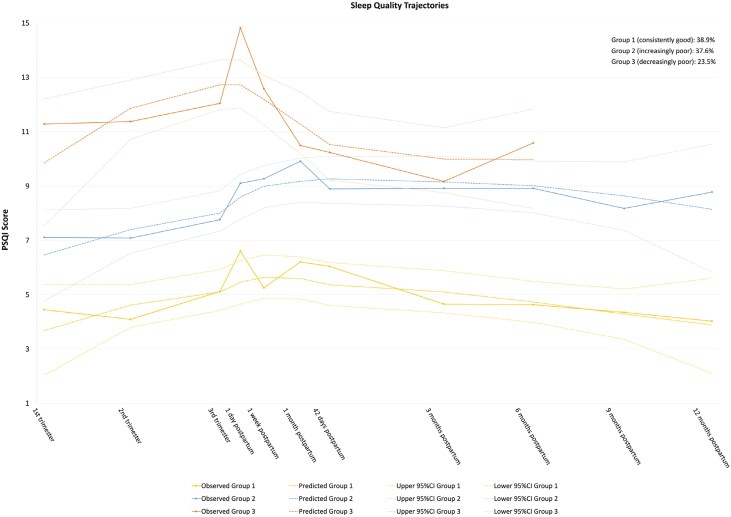
The quadratic three-trajectory model of sleep quality during the perinatal period.

To comprehensively analyze these trajectories [14–17, 20, 41–43], we extracted time points and corresponding PSQI scores from each trajectory and conducted a GBTM to classify sleep quality trajectories during the perinatal period. Results indicated that the quadratic three-trajectory model provided the optimal fit for the data (BIC = −224.98, AIC = −209.34, entropy = 0.951, Avepp = 0.957, 0.996, and 0.982; Figure 2). Group 1 remained relatively stable around a PSQI score of five throughout the perinatal period, accounting for 38.9%. Group 2 (37.6%) exhibited a slight increase in PSQI scores from the first trimester to 1 month postpartum, and then a gradual decline starting at 6 months postpartum. However, the scores persistently remained above five throughout. Group 3 (23.5%), the smallest and highest-scoring one, showed a clear increase in PSQI scores before 1 day postpartum and a significant decrease after that, though the scores remained above nine.

#### Sleep duration.

A total of four studies identified heterogeneous trajectories of sleep duration [18–21] (Figure S2). One study identified four distinct trajectories [18], two studies identified three distinct trajectories [19, 21], and the remaining one identified two trajectories [20]. Except for the long-increasing trajectory (31.6%) identified by Plancolaine et al., 2017 [19], all sleep duration trajectories exhibited a stable or declining trend. All studies investigated sleep duration across the first, second, and third trimesters, and identified one [20] or two trajectories [18, 19, 21] with sleep durations either below 6 or above 8 hours. The pooled prevalence of participants following an insufficient (<6 h) or excessive (>8 h) sleep duration trajectory was 22% (95% CI: 6% to 39%; I2 = 98.0%; 95% PI: 0% to 95.2%; Figure S6-A). None of the studies were classified as outliers or of low quality; therefore, sensitivity analyses were not performed. Subgroup meta-analysis by geographical region showed that the prevalence of nonoptimal sleep duration trajectory in Asia (13%) [18, 21] was lower than in North America (21%) [20] and Europe (43%) [19] (Figure S6-B).

#### Sleep efficiency.

Both Li et al., 2024 and Whitaker et al., 2021 observed two distinct sleep efficiency trajectories during pregnancy [20, 21] (Figure S3). They identified one stable high sleep efficiency trajectory [20, 21], with efficiency consistently around 90%, and both groups accounting for over 80% of the respective study populations. Li et al., 2024 identified a declining trajectory (11.6%), with efficiency decreasing from 83% in the first trimester to 73% in the third trimester [21]. In contrast, Whitaker et al., 2021 identified a stable trajectory (17.5%) that persistently remained at 67% [20]. The average prevalence of low sleep efficiency trajectory was 15%.

#### Other sleep dimensions.

One study investigated heterogeneous trajectories of sleep timing (e.g. bedtime and wake-up time) [21], while another focused on insomnia symptoms (Figure S4) [22]. Li et al., 2024 identified two distinct trajectories for bedtime (early, 49.1%; delayed, 50.9%) and wake-up time (early, 82.8%; late, 17.2%) across the three trimesters of pregnancy [21]. Sedov et al., 2020 identified three distinct trajectories for insomnia symptoms from early pregnancy to the early postpartum period, and 13.4% of perinatal women were classified into the clinical insomnia trajectory, characterized by ISI scores persistently above 14 during pregnancy and decreasing to 12 at 6 weeks postpartum [22]. Besides, women in the subclinical insomnia trajectory group (44.3%) experienced an increase in ISI scores before childbirth, followed by a marked decrease afterward. However, their ISI scores remained above seven throughout the perinatal period.

### Associated factors and health-related outcomes

A wide range of factors associated with sleep health trajectories was identified in 13 of the 17 studies (Table S5): demographics (age [18], race [20, 22], parity [18], and planned pregnancy [16]), socioeconomic factors (e.g. education [15, 16, 18, 20, 21], income [15], employment [17–19], occupational stress [41], and social support [14, 15, 41]), physical factors (health status [18], pre-pregnancy BMI [20, 21, 42], fatigue symptoms [14, 42], sleep quality [15, 16, 43]), and psychological factors (anxiety symptoms [15, 43, 46] and depressive symptoms [15, 16, 22, 41–43]). Maternal adverse pregnancy outcomes, including preterm birth [19, 21], assisted delivery [19], C-section [19], PPD [15, 16, 19, 22, 42], as well as postpartum mood disturbances [43], anxiety [22], fatigue [42] and excessive body weight gain [20, 42] were associated with sleep health trajectories. Moreover, significant differences in birth weight, length [21], and developmental outcomes [18] in infants were observed across distinct sleep health trajectory groups.

In particular, women with lower socioeconomic status (e.g. lower educational level, lower income, non-full-time employees, and lower social support) were more likely to fall into the persistently poor sleep quality trajectory, extremely long-decreasing sleep duration trajectory or late wake-up time trajectory, leading to higher risks of PPD [14–16] as well as low birth length and gross motor or language development delay in their infants [18, 21]. Poorer baseline sleep quality was significantly associated with a higher likelihood of following the persistently poor or worsening sleep quality trajectory, thus increasing the likelihood of PPD or postpartum mood disturbances [15, 16, 43]. Women with poorer self-reported health and higher fatigue symptoms at baseline have a higher risk of developing the trajectory of persistently poor sleep quality or short-decreasing sleep duration, which was in turn associated with an increased risk of PPD and overall development delay in their infants [14, 18]. A higher pre-pregnancy BMI was linked to the persistently poor sleep quality trajectory, subsequently increasing the likelihood of PPD, postpartum fatigue, and high BMI [42]. Besides, initial higher levels of anxiety or depressive symptoms were linked to the trajectories of persistently poor or worsening sleep quality and subclinical or clinical insomnia symptoms, further increasing the risk of PPD, postpartum anxiety, mood disturbances, fatigue, and high BMI [15, 16, 22, 42, 43]. Both insufficient (<6 hours) and excessive sleep duration (>8 hours) trajectories were linked to an elevated risk of adverse maternal outcomes (e.g. preterm birth, assisted delivery, C-section, and PPD), as well as low birth weight and development delay in infants (e.g. gross motor and language development) [18, 19]. The delayed bedtime trajectory was significantly associated with an increased likelihood of preterm birth [21].

## Discussion

### Summary of findings

To the best of our knowledge, this is the first systematic review and meta-analysis to comprehensively summarize trajectories of sleep health from pregnancy to the first year postpartum. We considered both single and heterogeneous trajectories across multiple dimensions of sleep health, including sleep quality, duration, efficiency, timing, and insomnia symptoms. Five studies focused on single sleep trajectories, which revealed the overall trends of sleep changes during the perinatal period. Additionally, 12 studies examining heterogeneous sleep trajectories identified two to four trajectory groups, and these trajectory groups differed in stability and severity. Overall, our findings highlight the heterogeneity of sleep health trajectories during the perinatal period, as well as the prevalence and associated factors of nonoptimal trajectories (e.g. low socioeconomic status, high pre-pregnancy BMI, poor sleep quality, poor self-reported health, and high levels of fatigue, anxiety, and depressive symptoms). Perinatal women with nonoptimal trajectories were more likely to develop adverse pregnancy outcomes and to experience developmental impacts on their infants.

In recent decades, advances in trajectory modeling techniques have led to a growing recognition of the importance of modeling sleep health trajectories to achieve a deeper understanding of population health dynamics and heterogeneity. An increasing number of studies have examined sleep health trajectories in children and adolescents [48, 49], but little is known about the trajectories of their mothers, particularly during the critical perinatal period. Considering the intergenerational relationship between maternal sleep and sleep trajectories of offspring [50], perinatal sleep health is likely to play a crucial role in shaping offspring sleep change patterns. Thus, future studies should place more emphasis on the sleep health trajectories of mothers during the perinatal period.

This is the first systematic review and meta-analysis to quantitatively synthesize the limited findings on perinatal sleep health trajectories. A total of 17 studies published in the past decade were included, highlighting that this is a relatively new and promising future research direction. Although findings on single sleep trajectories can provide an overview of perinatal sleep change patterns, they should be interpreted with caution due to the small sample sizes and limited representativeness of the included studies [8, 44–47]. Inconsistent findings from single sleep trajectory studies indicate the heterogeneity of perinatal sleep health trajectories, which suggests that considering the population as a whole without accounting for individual differences may not be a reasonable approach in real-world settings [51]. In light of these inconsistencies, there has been an increasing shift towards identifying distinct sleep health trajectories to capture the diverse nature of sleep development in perinatal women [14–22, 41–43]. We observed a spectrum of meaningful findings from these heterogeneous sleep trajectory studies, particularly in identifying perinatal women at the highest risk for adverse sleep health trajectories and associated outcomes.

A significant proportion of perinatal women experienced nonoptimal sleep health trajectories, with the highest prevalence observed in delayed bedtime (51%), followed by poor sleep quality (36%) and insufficient or excessive sleep duration (22%). Given that nonoptimal bedtime and sleep quality trajectories are more prevalent than sleep duration, future recommendations for improving perinatal sleep health should prioritize enhancing sleep quality and establishing earlier sleep routines. Our findings also reveal that geographical regions may be a significant source of heterogeneity in the prevalence of perinatal sleep health trajectories. Specifically, the prevalence of poor sleep quality trajectory was more prevalent among perinatal women in Asia compared to other regions, while their sleep duration appeared more optimal. This discrepancy could be attributed to cultural differences in work patterns and family structures [52, 53]. Further research is needed to better understand these regional disparities and their underlying causes.

We found that perinatal women with lower socioeconomic status were more likely to experience nonoptimal sleep quality, duration, and timing trajectories, which in turn heightened the risk of PPD [14–16], as well as low birth length and development delay in their infants [18, 21]. A recent study revealed that improvements in socioeconomic status were associated with better sleep duration trajectories [54]. Socioeconomic status has also been identified as an important moderator of the relationship between multiple sleep dimensions and health outcomes [55]. Thus, exploring the impact and underlying mechanisms of socioeconomic disparities on sleep health trajectories may help identify targeted sleep health interventions among disadvantaged groups, ultimately reducing the risk of adverse maternal and infant health outcomes. Besides, women with high pre-pregnancy BMI were more likely to follow a nonoptimal sleep quality trajectory, which has been associated with adverse postpartum health [42]. This highlights the need for causal studies to investigate the potential role of weight management on sleep quality trajectories and subsequent postpartum health. Furthermore, women with poorer sleep quality and self-reported health at baseline, as well as higher initial levels of fatigue, anxiety, and depressive symptoms were more likely to fall into nonoptimal sleep quality, duration, and insomnia symptoms trajectories, thereby increasing the risk of adverse postpartum health outcomes and development delay in their infants [14–16, 18, 19, 21, 22, 42, 43]. Therefore, integrating self-rated health screening tools (e.g. PSQI, Fatigue Continuum Form, State-Trait Anxiety Inventory, Edinburgh Postnatal Depression Scale) into routine prenatal care checkups could help identify high-risk women and address modifiable risk factors at an early stage.

Additionally, we identified three heterogeneous sleep quality trajectories (group 1: consistently good, 38.9%; group 2: increasingly poor, 37.6%; group 3: decreasingly poor, 23.5%) through GBTM based on eight studies, providing a general overview of perinatal sleep quality trends across diverse geographical regions. However, as six out of the eight studies were conducted in Asia, the generalizability of the findings remains limited. This also restricts further exploration of how cultural or regional factors shape perinatal sleep health trajectories. Future studies should be conducted in diverse cultural and geographical contexts to determine whether sleep health trajectories exhibit cultural or regional specificity. To enhance the comparability of findings, future research should provide comprehensive details on sample characteristics, particularly factors that may influence trajectory modeling results (e.g. parity and socioeconomic status). These factors should also be considered in trajectory analysis to enhance model accuracy and interpretability. It is noteworthy that a small but significant proportion of women (group 3) experienced improvements in sleep quality during the postpartum period, although their sleep quality did not reach an optimal level. This suggests that postpartum women may require interventions to restore optimal sleep health [56].

This review also highlights several areas in the sleep health trajectory study that merit further attention. First, the included studies of this review primarily focused on sleep quality and duration. Therefore, future research should place greater emphasis on other sleep health dimensions, such as efficiency, timing, regularity, insomnia symptoms, and daytime sleepiness, to provide a more comprehensive understanding of perinatal sleep health and their implications for maternal and offspring well-being. Second, all studies employed a subjective sleep measure to examine sleep health trajectories, which may introduce measurement bias, as subjective tools tend to overestimate or underestimate sleep health [57]. Future research should incorporate objective sleep measures, such as wearable electroencephalogram (EEG) sleep monitoring devices and actigraphy [58–60], to obtain a more nuanced understanding of sleep health trajectories. Third, most studies on sleep quality trajectories assessed sleep quality at only three-time points, with some studies using extremely short intervals, such as 1 day and 1 week postpartum. It raises questions about the consistency and clarity of guidance provided to participants when collecting sleep data at these specific time points because the PSQI reflects sleep over the past month. Thus, future studies should strategically set multiple time points across the perinatal period to ensure the precision of data collection and the robustness of trajectory identification. Fourth, the reporting quality of heterogeneous sleep health trajectory studies included in our review was generally low, and future studies should adhere to the criteria of GRoLTS. However, it was reassuring that almost all studies included in our review, including those involved in the GBTM and meta-analyses, were rated as good or fair quality based on the NOS. Finally, the investigation of predictors and the impact of nonoptimal sleep health trajectories on maternal and infant health outcomes remains inadequate. Future research should employ robust methodologies and explore a broader range of long-term health outcomes.

### Limitations

Our review, grounded in a longitudinal perspective, provides a unique insight into the understanding of sleep change patterns and the development of strategies to improve perinatal sleep health and well-being. A few limitations of this review should be noted. First, we implemented meta-analyses to pool the prevalence of poor sleep trajectories, despite the high heterogeneity. However, meta-analyses of prevalence frequently yield high I^2^. Therefore, we estimated prediction intervals to explore heterogeneity tried to perform subgroup analyses to explore the sources of heterogeneity, and conducted sensitivity analysis to mitigate the risk of producing spurious findings. Second, we extracted time points and corresponding PSQI scores from trajectory plots in the included studies to generate a new trajectory model for sleep quality. Combining these data quantitatively poses risks due to significant missing values. Therefore, we opted for the GBTM approach, which can yield unbiased estimates in the presence of missing data. Additionally, we ensured the number of observations exceeded 100 to maintain the statistical power of GBTM. Although GBTM is a powerful method, its application in this context should be viewed with caution and future research with more comprehensive datasets is needed to further validate these results. Another limitation is that all the included studies were conducted in upper- or middle-income countries, which limits the generalizability of the findings to low-income countries. Moreover, the sample was relatively younger, healthier, and predominantly with higher socioeconomic status, further restricting the generalizability of the findings. Future studies should be conducted in low-income countries to address this gap and include more diverse and representative samples to enhance it.

## Conclusion

This review provides a clearer understanding of the prevalence, distribution, and burden of sleep health disparities among perinatal women from a longitudinal perspective. It highlights the heterogeneity of sleep health trajectories during pregnancy to 1 year postpartum, and quantitively synthesizes the findings of these trajectories for the first time. The pooled prevalence of women in poor sleep quality trajectories was 36%, and the pooled prevalence of women in insufficient or excessive sleep duration trajectories was 22%. The mean prevalence of low sleep efficiency trajectory was 15%, while the prevalence of delayed bedtime, late wake-up time, and clinical insomnia trajectories was reported as 51%, 17%, and 13%, respectively. Moreover, a three-group trajectory model was re-identified for sleep quality: group 1 (consistently good, 38.9%), group 2 (increasingly poor, 37.6%), and group 3 (decreasingly poor, 23.5%). Nonoptimal sleep health trajectories were associated with higher risks of adverse maternal and infant outcomes, and socioeconomic status, pre-pregnancy BMI, self-reported health, fatigue, anxiety, and depressive symptoms may serve as important predictors of these high-risk trajectories. These findings may provide valuable insights for informing future research and guiding the development of targeted strategies to enhance perinatal sleep health among high-risk trajectory groups, thereby potentially reducing sleep health disparities and contributing to improved maternal and infant outcomes.

## Supplementary material

Supplementary material is available at *SLEEP* online.

zsaf095_suppl_Supplementary_Tables_S1-S5_Figures_S1-S6

## Data Availability

Data and ana data code were provided in the supplementary material.
