# Supplementary material for: Trajectories of sleep health during the perinatal period: a systematic review and meta-analysis
Source: Sleep. 2025 Apr 28;48(7):zsaf095. doi: 10.1093/sleep/zsaf095 (PMC12246372; doi:10.1093/sleep/zsaf095)

**Trajectories of sleep health during the perinatal period: A systematic review and meta-analysis**

**Authors**

Man Wang^1, 2^, Jialu Qian^3^, Youngmin Cho^2^, Zhiting Guo^1, 2^, Xiaoyan Yu^4*^, Junxin Li^2^

**Affiliations**

^1^Zhejiang University School of Medicine, Hangzhou, Zhejiang, China

^2^Johns Hopkins University School of Nursing, Baltimore, Maryland, the United States

^3^Nanjing University of Chinese Medicine School of Nursing, Nanjing, Jiangsu, China

^4^Women’s Hospital School of Medicine Zhejiang University, Hangzhou, Zhejiang, China

**Corresponding Author**

^*^Xiaoyan Yu, Women’s Hospital School of Medicine Zhejiang University, 1 Xueshi Rd, Shangcheng District, Hangzhou, Zhejiang 310002, China, e-mail address: yuxy@zju.edu.cn

**Captions for supplementary tables and figures**

Supplementary Table S1 Search strategy

Supplementary Table S2 Data extracted from studies examine sleep quality trajectories

Supplementary Table S3 Study quality assessment based on the Newcastle–Ottawa scale (NOS)

Supplementary Table S4 Assessment of latent trajectory analysis based on the Guidelines for reporting on latent trajectory studies (GRoLTS)

Supplementary Table S5 Associated factors and health-related outcomes of sleep health trajectories

Supplementary Figure S1 Heterogenous trajectories of sleep quality (n = 8)

Supplementary Figure S2 Heterogenous trajectories of sleep duration (n = 4)

Supplementary Figure S3 Heterogenous trajectories of sleep efficiency (n = 2)

Supplementary Figure S4 Heterogenous trajectories of sleep timing and insomnia symptoms (n = 2)

Supplementary Figure S5 Meta-analysis of the prevalence of poor sleep quality trajectories (PSQI > 5) (n = 6)

Supplementary Figure S6 Meta-analysis of the prevalence of insufficient (< 6h) or excessive (> 8h) sleep duration trajectories (n = 4)

**Supplementary Table S1 Search strategy**

| **Databases** |  | **Combinations of search terms** | **Quantity** |
| --- | --- | --- | --- |
| Embase | #1 | 'pregnant woman'/exp OR 'pregnancy'/exp OR 'puerperium'/exp OR 'perinatal period'/exp OR 'prenatal period'/exp OR 'expectant mother'/exp | 1,094,326 |
|  | #2 | postpartum:ab,ti OR trimester*:ab,ti OR gestation:ab,ti OR antenatal:ab,ti OR postnatal:ab,ti OR puerper*:ab,ti OR maternal:ab,ti OR antepartum:ab,ti OR peripartum:ab,ti | 866,900 |
|  | #3 | #1 OR #2 | 1,518,159 |
|  | #4 | 'sleep'/exp OR 'sleep disorder'/exp OR 'sleep parameters'/exp | 528,341 |
|  | #5 | 'sleep duration':ab,ti OR bedtime:ab,ti OR 'wake-up time':ab,ti OR 'sleep quantity':ab,ti OR sleepiness:ab,ti OR 'sleep opportunit*':ab,ti OR 'insomnia symptom*':ab,ti OR 'sleep quality':ab,ti OR 'sleep time':ab,ti OR somnolence:ab,ti OR 'sleep continuity':ab,ti OR 'sleep timing':ab,ti OR alertness:ab,ti OR napping:ab,ti OR 'sleep satisfaction':ab,ti OR 'sleep stage*':ab,ti OR 'sleep efficiency':ab,ti OR 'sleep latency':ab,ti | 137,865 |
|  | #6 | #4 OR #5 | 547,166 |
|  | #7 | 'growth curve'/exp | 24,960 |
|  | #8 | trajector*:ab,ti OR change*:ab,ti OR longitudinal:ab,ti OR cohort*:ab,ti OR 'follow up*':ab,ti OR pattern*:ab,ti OR group*:ab,ti OR development*:ab,ti | 16,663,109 |
|  | #9 | #7 OR #8 | 16,673,681 |
|  | #10 | #3 AND #6 AND #9 | 9,934 |
|  | #11 | #8 AND [english]/lim | 9,563 |
| Scopus | 1 | TITLE ( "pregnant wom*n" OR "pregnanc*" OR postpartum OR "trimester*" OR gestation OR perinatal OR antenatal OR postnatal OR prenatal OR maternal OR "mother*" OR "puerper*" OR "antepartum" OR "peripartum" ) | 669,633 |
|  | 2 | ABS ( "pregnant wom*n" OR "pregnanc*" OR postpartum OR "trimester*" OR gestation OR perinatal OR antenatal OR postnatal OR prenatal OR maternal OR "mother*" OR "puerper*" OR "antepartum" OR "peripartum" ) | 1,299,335 |
|  | 3 | 1 OR 2 | 1,500,771 |
|  | 4 | TITLE ( sleep OR "sleep wake disorder*" OR "sleep stage*" OR "sleep duration" OR "sleep time" OR bedtime OR "wake-up time" OR "sleep quantity" OR "sleep efficiency" OR "sleep continuity" OR "sleep timing" OR alertness OR somnolence OR sleepiness OR napping OR "sleep satisfaction" OR "sleep quality" OR "sleep latency" OR "sleep opportunit*" OR "insomnia symptom*" ) | 159,209 |
|  | 5 | ABS ( sleep OR "sleep wake disorder*" OR "sleep stage*" OR "sleep duration" OR "sleep time" OR bedtime OR "wake-up time" OR "sleep quantity" OR "sleep efficiency" OR "sleep continuity" OR "sleep timing" OR alertness OR somnolence OR sleepiness OR napping OR "sleep satisfaction" OR "sleep quality" OR "sleep latency" OR "sleep opportunit*" OR "insomnia symptom*" ) | 293,470 |
|  | 6 | 4 OR 5 | 325,284 |
|  | 7 | TITLE ( "trajector*" OR "change*" OR longitudinal OR "cohort*" OR "follow-up*" OR "pattern*" OR "group*" OR "development*" OR "growth curve" ) | 4,580,298 |
|  | 8 | ABS ( "trajector*" OR "change*" OR longitudinal OR "cohort*" OR "follow-up*" OR "pattern*" OR "group*" OR "development*" OR "growth curve" ) | 26,223,241 |
|  | 9 | 7 OR 8 | 27,688,579 |
|  | 10 | 3 AND 6 AND 9 | 7,695 |
|  | 11 | 10 AND ( LIMIT-TO ( LANGUAGE , "english" ) ) | 7,037 |
| Web of Science | #1 | TI=(pregnant wom*n OR pregnanc* OR postpartum OR trimester* OR gestation OR perinatal OR antenatal OR postnatal OR prenatal OR maternal OR mother* OR puerper* OR antepartum OR peripartum) | 655,373 |
|  | #2 | AB=(pregnant wom*n OR pregnanc* OR postpartum OR trimester* OR gestation OR perinatal OR antenatal OR postnatal OR prenatal OR maternal OR mother* OR puerper* OR antepartum OR peripartum) | 1,014,077 |
|  | #3 | #1 OR #2 | 1,296,143 |
|  | #4 | TI=(sleep OR sleep wake disorder* OR sleep stage* OR sleep duration OR sleep time OR bedtime OR wake-up time OR sleep quantity OR sleep efficiency OR sleep continuity OR sleep timing OR alertness OR somnolence OR sleepiness OR napping OR sleep satisfaction OR sleep quality OR sleep latency OR sleep opportunit* OR insomnia symptom*) | 196,643 |
|  | #5 | AB=(sleep OR sleep wake disorder* OR sleep stage* OR sleep duration OR sleep time OR bedtime OR wake-up time OR sleep quantity OR sleep efficiency OR sleep continuity OR sleep timing OR alertness OR somnolence OR sleepiness OR napping OR sleep satisfaction OR sleep quality OR sleep latency OR sleep opportunit* OR insomnia symptom*) | 261,798 |
|  | #6 | #4 OR #5 | 350,895 |
|  | #7 | TI=(trajector* OR change* OR longitudinal OR cohort* OR follow-up* OR pattern* OR group* OR development* OR growth curve) | 4,492,288 |
|  | #8 | AB=(trajector* OR change* OR longitudinal OR cohort* OR follow-up* OR pattern* OR group* OR development* OR growth curve) | 19,396,628 |
|  | #9 | #7 OR #8 | 21,497,465 |
|  | #10 | #3 AND #6 AND #9 | 7,231 |
|  | #11 | #10 AND (LA=("ENGLISH")) | 6,971 |
| PubMed | #1 | "Pregnant Women"[MeSH Terms] OR "Pregnancy"[MeSH Terms] OR "Postpartum Period"[MeSH Terms] OR "Pregnancy Trimesters"[MeSH Terms] OR "Peripartum Period"[MeSH Terms] | 1,066,852 |
|  | #2 | "gestation"[Title/Abstract] OR "perinatal"[Title/Abstract] OR "antenatal"[Title/Abstract] OR "postnatal"[Title/Abstract] OR "prenatal"[Title/Abstract] OR "maternal"[Title/Abstract] OR "mother*"[Title/Abstract] OR "puerper*"[Title/Abstract] OR "antepartum"[Title/Abstract] OR "peripartum"[Title/Abstract] | 832,931 |
|  | #3 | #1 OR #2 | 1,481,732 |
|  | #4 | "Sleep"[MeSH Terms] OR "Sleep Wake Disorders"[MeSH Terms] | 180,959 |
|  | #5 | "sleep duration"[Title/Abstract] OR "sleep time"[Title/Abstract] OR "bedtime"[Title/Abstract] OR "wake-up time"[Title/Abstract] OR "sleep quantity"[Title/Abstract] OR "sleep efficiency"[Title/Abstract] OR "sleep continuity"[Title/Abstract] OR "sleep timing"[Title/Abstract] OR "alertness"[Title/Abstract] OR "somnolence"[Title/Abstract] OR "sleepiness"[Title/Abstract] OR "napping"[Title/Abstract] OR "sleep satisfaction"[Title/Abstract] OR "sleep quality"[Title/Abstract] OR "sleep latency"[Title/Abstract] OR "sleep opportunit*"[Title/Abstract] OR "insomnia symptom*"[Title/Abstract] OR "sleep stage*"[ Title/Abstract] | 85,597 |
|  | #6 | #4 OR #5 | 215,587 |
|  | #7 | "trajector*"[Title/Abstract] OR "change*"[Title/Abstract] OR "development*"[Title/Abstract] OR "longitudinal"[Title/Abstract] OR "cohort*"[Title/Abstract] OR "follow-up*"[Title/Abstract] OR "pattern*"[Title/Abstract] OR "group*"[Title/Abstract] OR "growth curve"[Title/Abstract] | 12,295,608 |
|  | #8 | #3 AND #6 AND #7 | 4,592 |
|  | #9 | #8 AND (english[Filter]) | 4,357 |
| CINAHL | S1 | (MH "Pregnancy") OR (MH "Pregnancy Trimesters") OR (MH "Postnatal Period") OR (MH "Expectant Mothers") | 239,014 |
|  | S2 | TI (pregnant wom*n OR postpartum OR gestation OR antenatal OR prenatal OR perinatal OR maternal OR puerper* OR antepartum OR peripartum) | 171,266 |
|  | S3 | AB (pregnant wom*n OR postpartum OR gestation OR antenatal OR prenatal OR perinatal OR maternal OR puerper* OR antepartum OR peripartum) | 208,809 |
|  | S4 | S1 OR S2 OR S3 | 366,423 |
|  | S5 | (MH "Sleep") OR (MH "Sleep Disorders") OR (MH "Sleepiness") | 36,742 |
|  | S6 | TI (sleep time OR bedtime OR wake-up time OR sleep quantity OR sleep efficiency OR sleep continuity OR sleep timing OR alertness OR somnolence OR napping OR sleep satisfaction OR sleep opportunit* OR sleep stages OR sleep duration OR sleep latency OR sleep quality OR insomnia symptom*) | 9,333 |
|  | S7 | AB (sleep time OR bedtime OR wake-up time OR sleep quantity OR sleep efficiency OR sleep continuity OR sleep timing OR alertness OR somnolence OR napping OR sleep satisfaction OR sleep opportunit* OR sleep stages OR sleep duration OR sleep latency OR sleep quality OR insomnia symptom*) | 26,830 |
|  | S8 | S5 OR S6 OR S7 | 54,288 |
|  | S9 | TI (trajector* OR change* OR longitudinal OR cohort* OR follow-up* OR pattern* OR group* OR development* OR growth curve) | 506,443 |
|  | S10 | AB (trajector* OR change* OR longitudinal OR cohort* OR follow-up* OR pattern* OR group* OR development* OR growth curve) | 2,040,458 |
|  | S11 | S9 OR S10 | 2,277,685 |
|  | S12 | S4 AND S8 AND S11 | 1,373 |
|  | S13 | Narrow by Language: - english | 1,339 |
| PsycINFO | S1 | (MH "Pregnancy") OR (MH "Pregnancy Trimesters") OR (MH "Postnatal Period") OR (MH "Expectant Mothers") | 5 |
|  | S2 | TI (pregnant wom*n OR postpartum OR gestation OR antenatal OR prenatal OR perinatal OR maternal OR puerper* OR antepartum OR peripartum) | 61,160 |
|  | S3 | AB (pregnant wom*n OR postpartum OR gestation OR antenatal OR prenatal OR perinatal OR maternal OR puerper* OR antepartum OR peripartum) | 138,632 |
|  | S4 | S1 OR S2 OR S3 | 144,312 |
|  | S5 | (MH "Sleep") OR (MH "Sleep Disorders") OR (MH "Sleepiness") | 4 |
|  | S6 | TI (sleep time OR bedtime OR wake-up time OR sleep quantity OR sleep efficiency OR sleep continuity OR sleep timing OR alertness OR somnolence OR napping OR sleep satisfaction OR sleep opportunit* OR sleep stages OR sleep duration OR sleep latency OR sleep quality OR insomnia symptom*) | 8,143 |
|  | S7 | AB (sleep time OR bedtime OR wake-up time OR sleep quantity OR sleep efficiency OR sleep continuity OR sleep timing OR alertness OR somnolence OR napping OR sleep satisfaction OR sleep opportunit* OR sleep stages OR sleep duration OR sleep latency OR sleep quality OR insomnia symptom*) | 40,624 |
|  | S8 | S5 OR S6 OR S7 | 41,259 |
|  | S9 | TI (trajector* OR change* OR longitudinal OR cohort* OR follow-up* OR pattern* OR group* OR development* OR growth curve) | 501,723 |
|  | S10 | AB (trajector* OR change* OR longitudinal OR cohort* OR follow-up* OR pattern* OR group* OR development* OR growth curve) | 2,475,029 |
|  | S11 | S9 OR S10 | 2,566,776 |
|  | S12 | S4 AND S8 AND S11 | 984 |
|  | S13 | Narrow by Language: - english | 964 |
| Wanfang Data | 1 | 主题:(孕妇) or 主题:(产妇) or 主题:(怀孕) or 主题:(分娩) or 主题:(孕产妇) or 主题:(妊娠期) or 主题:(产褥期) or 主题:(围产期) or 主题:(围生期) or 主题:(产前) or 主题:(产后) or 主题:(孕早期) or 主题:(孕中期) or 主题:(孕晚期) | 591,840 |
|  | 2 | 主题:(睡眠) or 主题:(睡眠障碍) or 主题:(睡眠阶段) or 主题:(睡眠时间) or 主题:(起床时间) or 主题:(入睡时间) or 主题:(睡眠效率) or 主题:(睡眠规律) or 主题:(警觉性) or 主题:(嗜睡) or 主题:(午睡) or 主题:(睡眠满意度) or 主题:(睡眠质量) or 主题:(睡眠潜伏期) or 主题:(失眠症状) | 207,599 |
|  | 3 | 主题:(轨迹) or 主题:(发展) or 主题:(变化) or 主题:(模式) or 主题:(纵向) or 主题:(队列) or 主题:(随访) or 主题:(亚组) or 主题:(类别) or 主题:(增长曲线) | 23,590,789 |
|  | 4 | 1 and 2 and 3  限制：期刊论文；专业检索；主题词扩展；中文 | 1400 |
| CNKI | 1 | SU%=孕妇 OR SU%=产妇 OR SU%=怀孕 OR SU%=分娩 OR SU%=孕产期 OR SU%=妊娠期 OR SU%=产褥期 OR SU%=围产期 OR SU%=围生期 OR SU%=产前 OR SU%=产后 OR SU%=孕早期 OR SU%=孕中期 OR SU%=孕晚期 | 617,658 |
|  | 2 | SU%=睡眠 OR SU%=睡眠障碍 OR SU%=睡眠阶段 OR SU%=睡眠时间 OR SU%=起床时间 OR SU%=入睡时间 OR SU%=睡眠效率 OR SU%=睡眠规律 OR SU%=警觉性 OR SU%=嗜睡 OR SU%=午睡 OR SU%=睡眠满意度 OR SU%=睡眠质量 OR SU%=睡眠潜伏期OR SU%=失眠症状 | 152,124 |
|  | 3 | SU%=轨迹 OR SU%=发展 OR SU%=变化 OR SU%=模式 OR SU%=纵向 OR SU%=队列 OR SU%=随访 OR SU%=亚组 OR SU%=类别 OR SU%=增长曲线 | 17,794,824 |
|  | 4 | 1 AND 2 OR 3  限制：专业检索；同义词扩展；学术期刊；中文 | 202 |

Note: The searches were conducted on August 13, 2024.

**Supplementary Table S2 Data extracted from studies examine sleep quality trajectories**

| **ID** | **PSQI1** | **PSQI2** | **PSQI3** | **PSQI4** | **PSQI5** | **PSQI6** | **PSQI7** | **PSQI8** | **PSQI9** | **PSQI10** | **PSQI11** | **Time1** | **Time2** | **Time3** | **Time4** | **Time5** | **Time6** | **Time7** | **Time8** | **Time9** | **Time10** | **Time11** |
| --- | --- | --- | --- | --- | --- | --- | --- | --- | --- | --- | --- | --- | --- | --- | --- | --- | --- | --- | --- | --- | --- | --- |
| Zhang 2023-1 |  |  | 14.70146 | 15.46222 | 13.49641 |  |  |  |  |  |  | 1 | 2 | 3 | 4 | 5 | 6 | 7 | 8 | 9 | 10 | 11 |
| Zhang 2023-2 |  |  | 5.25581 | 8.45711 | 12.9304 |  |  |  |  |  |  | 1 | 2 | 3 | 4 | 5 | 6 | 7 | 8 | 9 | 10 | 11 |
| Zhang 2023-3 |  |  | 6.80777 | 7.69634 | 6.17481 |  |  |  |  |  |  | 1 | 2 | 3 | 4 | 5 | 6 | 7 | 8 | 9 | 10 | 11 |
| Tzeng 2015-1 |  |  | 13.58881 | 14.20186 | 14.56968 | 10.76878 |  |  | 10.72791 |  |  | 1 | 2 | 3 | 4 | 5 | 6 | 7 | 8 | 9 | 10 | 11 |
| Tzeng 2015-2 |  |  | 8.60267 | 10.11486 | 9.91051 | 9.62442 |  |  | 7.37658 |  |  | 1 | 2 | 3 | 4 | 5 | 6 | 7 | 8 | 9 | 10 | 11 |
| Tzeng 2015-3 |  |  | 5.45569 | 5.90526 | 5.33308 | 6.84527 |  |  | 5.21047 |  |  | 1 | 2 | 3 | 4 | 5 | 6 | 7 | 8 | 9 | 10 | 11 |
| Tomfohr 2015-1 |  | 11.89803 | 9.15312 |  |  |  |  | 8.9297 | 10.46174 |  |  | 1 | 2 | 3 | 4 | 5 | 6 | 7 | 8 | 9 | 10 | 11 |
| Tomfohr 2015-2 |  | 6.72738 | 10.11065 |  |  |  |  | 10.3979 | 10.04681 |  |  | 1 | 2 | 3 | 4 | 5 | 6 | 7 | 8 | 9 | 10 | 11 |
| Tomfohr 2015-3 |  | 4.97192 | 5.99328 |  |  |  |  | 5.99328 | 5.83369 |  |  | 1 | 2 | 3 | 4 | 5 | 6 | 7 | 8 | 9 | 10 | 11 |
| Tomfohr 2015-4 |  | 3.6633 | 3.47179 |  |  |  |  | 2.70577 | 2.73769 |  |  | 1 | 2 | 3 | 4 | 5 | 6 | 7 | 8 | 9 | 10 | 11 |
| Lin-Lewry 2023-1 |  | 11.05216 | 12.16453 |  | 11.35554 | 10.26845 |  | 9.43417 |  |  |  | 1 | 2 | 3 | 4 | 5 | 6 | 7 | 8 | 9 | 10 | 11 |
| Lin-Lewry 2023-2 |  | 6.19819 | 7.05774 |  | 9.00439 | 10.26845 |  | 7.84146 |  |  |  | 1 | 2 | 3 | 4 | 5 | 6 | 7 | 8 | 9 | 10 | 11 |
| Lin-Lewry 2023-3 |  | 4.04929 | 4.90885 |  | 5.16166 | 5.56616 |  | 5.03525 |  |  |  | 1 | 2 | 3 | 4 | 5 | 6 | 7 | 8 | 9 | 10 | 11 |
| Wang 2018-1 |  |  | 8.34229 |  |  |  | 9.21242 | 8.54309 | 9.30166 | 8.20842 | 8.78851 | 1 | 2 | 3 | 4 | 5 | 6 | 7 | 8 | 9 | 10 | 11 |
| Wang 2018-2 |  |  | 5.91038 |  |  |  | 7.73989 | 6.10043 | 5.86576 | 5.64265 | 5.1295 | 1 | 2 | 3 | 4 | 5 | 6 | 7 | 8 | 9 | 10 | 11 |
| Wang 2018-3 |  |  | 3.7239 |  |  |  | 5.19643 | 3.5231 | 3.61235 | 3.07688 | 2.94301 | 1 | 2 | 3 | 4 | 5 | 6 | 7 | 8 | 9 | 10 | 11 |
| Whitaker 2021-1 | 11.2864 | 11.33015 | 11.2864 |  |  |  |  |  |  |  |  | 1 | 2 | 3 | 4 | 5 | 6 | 7 | 8 | 9 | 10 | 11 |
| Whitaker 2021-2 | 6.05927 | 7.80894 | 9.58048 |  |  |  |  |  |  |  |  | 1 | 2 | 3 | 4 | 5 | 6 | 7 | 8 | 9 | 10 | 11 |
| Whitaker 2021-3 | 5.03135 | 4.13464 | 5.42502 |  |  |  |  |  |  |  |  | 1 | 2 | 3 | 4 | 5 | 6 | 7 | 8 | 9 | 10 | 11 |
| Lyu 2020-1 | 8.15 | 7.3 | 7.79 |  |  |  |  |  |  |  |  | 1 | 2 | 3 | 4 | 5 | 6 | 7 | 8 | 9 | 10 | 11 |
| Lyu 2020-2 | 3.84 | 3.71 | 5.56 |  |  |  |  |  |  |  |  | 1 | 2 | 3 | 4 | 5 | 6 | 7 | 8 | 9 | 10 | 11 |
| Bao 2022-1 |  |  | 11.2636 |  | 11.3873 |  | 10.27404 |  |  |  |  | 1 | 2 | 3 | 4 | 5 | 6 | 7 | 8 | 9 | 10 | 11 |
| Bao 2022-2 |  |  | 5.61483 |  | 6.27454 |  | 11.13991 |  |  |  |  | 1 | 2 | 3 | 4 | 5 | 6 | 7 | 8 | 9 | 10 | 11 |
| Bao 2022-3 |  |  | 7.88258 |  | 9.16077 |  | 6.39823 |  |  |  |  | 1 | 2 | 3 | 4 | 5 | 6 | 7 | 8 | 9 | 10 | 11 |
| Bao 2022-4 |  |  | 4.37787 |  | 4.62526 |  | 5.16127 |  |  |  |  | 1 | 2 | 3 | 4 | 5 | 6 | 7 | 8 | 9 | 10 | 11 |

Note: PSQI = Pittsburgh sleep quality index; PSQI1 to PSQI11 refer to PSQI scores at Time 1 to Time 12, respectively. Time1 = 1st trimester (1); Time2 = 2nd trimester (2); Time3 = 3rd trimester (3); Time4 = 1 day postpartum (4); Time5 = 1 week postpartum (5); Time6 = 1 month postpartum (6); Time7 = 42 days postpartum (7); Time8 = 3 months postpartum (8); Time9 = 6 months postpartum (9); Time10 = 9 months postpartum (10); Time11 = 12 months postpartum (11). The data analysis was conducted using SAS 9.4, and the relevant code is provided below.

Code (SAS 9.4):

PROC IMPORT DATAFILE="C:\Users\wmzxf\Desktop\Data.xlsx"

OUT=work.mydata

DBMS=xlsx

REPLACE;

RUN;

PROC TRAJ DATA=mydata OUTPLOT=OP OUTSTAT=OS OUT=OF OUTEST=OE ITDETAIL;

ID ID; VAR PSQI1-PSQI11; INDEP Time1-Time11;

MODEL CNORM;MAX 21; NGROUPS 3; ORDER 2 2 2;

RUN;

PROC SGPLOT DATA=OP;

SERIES X=Time Y=PSQI / GROUP=GROUP;

XAXIS LABEL="Time";

YAXIS LABEL="Predicted PSQI";

TITLE "Trajectory Analysis";

RUN;

**Supplementary Table S3 Study quality assessment based on the Newcastle–Ottawa scale (NOS)**

| **Author** | **Study type** | **Selection** | | | | **Comparability** | **Outcome** | | | **Total quality** |
| --- | --- | --- | --- | --- | --- | --- | --- | --- | --- | --- |
|  |  | 1 | 2 | 3 | 4 | 5 | 6 | 7 | 8 |  |
| Zhang et al., 2023 [41] | Longitudinal study | 🟊 | 🟊 |  | NA | NA | NA | NA | 🟊 | Good |
| Tomfohr et al., 2015 [15] | Cohort study | 🟊 | 🟊 |  | 🟊 | 🟊🟊 |  | 🟊 | 🟊 | Good |
| Tzeng et al., 2015 [39] | Cohort study | 🟊 | 🟊 |  | 🟊 | 🟊 |  | 🟊 | 🟊 | Good |
| Sedov et al., 2020 [22] | Cohort study | 🟊 | 🟊 |  | 🟊 |  |  | 🟊 | 🟊 | Poor |
| Lin-Lewry et al., 2023 [14] | Cohort study | 🟊 | 🟊 |  |  | 🟊🟊 |  | 🟊 | 🟊 | Fair |
| Wang et al., 2018 [43] | Cohort study | 🟊 | 🟊 |  | 🟊 | 🟊🟊 |  | 🟊 | 🟊 | Good |
| Whitaker et al., 2021 [20] | Cohort study | 🟊 | 🟊 |  | 🟊 | 🟊🟊 |  | 🟊 | 🟊 | Good |
| Plancoulaine et al., 2017 [19] | Cohort study | 🟊 | 🟊 |  | 🟊 | 🟊🟊 |  | 🟊 | 🟊 | Good |
| Lyu et al., 2020 [17] | Cohort study | 🟊 | 🟊 |  | 🟊 | 🟊🟊 |  | 🟊 | 🟊 | Good |
| Bao et al., 2022 [16] | Cohort study | 🟊 | 🟊 |  | 🟊 | 🟊🟊 |  | 🟊 | 🟊 | Good |
| Li et al., 2023 [18] | Cohort study | 🟊 | 🟊 |  | 🟊 | 🟊🟊 | 🟊 | 🟊 | 🟊 | Good |
| Li et al., 2024 [21] | Cohort study | 🟊 | 🟊 |  | 🟊 | 🟊🟊 | 🟊 | 🟊 | 🟊 | Good |
| van der Zwan et al., 2017 [46] | Cohort study | 🟊 | 🟊 |  |  | 🟊🟊 |  | 🟊 |  | Poor |
| Paul et al., 2019 [47] | Longitudinal study | 🟊 | 🟊 |  | NA | NA | NA | NA | 🟊 | Good |
| Verma et al., 2024 [44] | Longitudinal study | 🟊 | 🟊 |  | NA | NA | NA | NA | 🟊 | Good |
| Horwitz et al., 2023 [8] | Longitudinal study | 🟊 | 🟊 | 🟊 | NA | NA | NA | NA | 🟊 | Good |
| Nagi et al., 2015 [45] | Longitudinal study | 🟊 | 🟊 |  | NA | NA | NA | NA | 🟊 | Good |

Note: 1=Representativeness of exposed cohort, 2=Selection of non-exposed cohort, 3=Exposure ascertainment, 4=Outcome was not present at the start, 5=Confounders controlled, 6=Assessment of outcome, 7=Follow-up long enough, 8=Adequacy of follow-up (follow-up rate < 50%). NA = Not available. Longitudinal studies refer to studies that focus solely on changes in sleep over time during the perinatal period. Cohort studies refer to studies that not only examine sleep changes during the perinatal period but also explore their longitudinal associations with maternal or infant outcomes. For cohort studies, quality was classified as follows: 1) good quality: 3 or 4 stars in the selection domain, 1 or 2 stars in the comparability domain, and 2 or 3 stars in the outcome domain; 2) fair quality: 2 stars in the selection domain, 1 or 2 stars in the comparability domain, and 2 or 3 stars in the outcome domain; and 3) poor quality: 0 or 1 star in the selection domain, 0 stars in the comparability domain, or 0 or 1 star in the outcome domain. For longitudinal studies, quality was classified as follows: 1) good quality: 2 or 3 stars in the selection domain and 1 star in the outcome domain; 2) fair quality: 1 star in both the selection and outcome domains; and 3) poor quality: 0 stars in either the selection or outcome domain.

**Supplementary Table S4 Assessment of latent trajectory analysis based on the** **Guidelines for reporting on latent trajectory studies (GRoLTS)**

| **Author** | **A** | **B** | **C1** | **C2** | **C3** | **D** | **E** | **F1** | **F2** | **G** | **H** | **I** | **J** | **K** | **L** | **M** | **N1** | **N2** | **N3** | **O** | **P** | **Total score** |
| --- | --- | --- | --- | --- | --- | --- | --- | --- | --- | --- | --- | --- | --- | --- | --- | --- | --- | --- | --- | --- | --- | --- |
| Zhang et al., 2023 [41] | 1 | 0 | 0 | 0 | 0 | 0 | 1 | 0 | 0 | 0 | 0 | 0 | 1 | 1 | 1 | 1 | 1 | 0 | 0 | 1 | 0 | 8 |
| Tomfohr et al., 2015 [15] | 1 | 1 | 1 | 1 | 1 | 0 | 1 | 0 | 0 | 0 | 0 | 0 | 1 | 1 | 0 | 0 | 1 | 0 | 0 | 1 | 0 | 10 |
| Tzeng et al., 2015 [39] | 1 | 0 | 0 | 0 | 0 | 0 | 1 | 0 | 0 | 0 | 0 | 0 | 1 | 0 | 0 | 0 | 1 | 0 | 0 | 0 | 0 | 4 |
| Sedov et al., 2020 [22] | 1 | 1 | 1 | 1 | 1 | 0 | 1 | 0 | 0 | 1 | 0 | 0 | 1 | 1 | 0 | 0 | 1 | 0 | 0 | 1 | 0 | 11 |
| Lin-Lewry et al., 2023 [14] | 1 | 0 | 1 | 1 | 0 | 0 | 1 | 0 | 0 | 0 | 0 | 0 | 1 | 1 | 0 | 0 | 1 | 0 | 0 | 0 | 0 | 7 |
| Wang et al., 2018 [43] | 1 | 0 | 1 | 1 | 1 | 1 | 1 | 0 | 0 | 0 | 0 | 0 | 1 | 1 | 0 | 0 | 1 | 0 | 0 | 0 | 0 | 9 |
| Whitaker et al., 2021 [20] | 1 | 0 | 1 | 0 | 1 | 0 | 1 | 0 | 0 | 0 | 0 | 0 | 1 | 0 | 0 | 0 | 1 | 0 | 0 | 0 | 0 | 6 |
| Plancoulaine et al., 2017 [19] | 1 | 0 | 1 | 1 | 1 | 0 | 1 | 0 | 0 | 0 | 0 | 0 | 1 | 0 | 0 | 0 | 1 | 0 | 0 | 0 | 0 | 7 |
| Lyu et al., 2020 [17] | 1 | 1 | 1 | 0 | 1 | 0 | 1 | 0 | 0 | 0 | 0 | 0 | 1 | 1 | 0 | 0 | 1 | 0 | 0 | 1 | 0 | 9 |
| Bao et al., 2022 [16] | 1 | 0 | 0 | 1 | 0 | 0 | 1 | 0 | 0 | 0 | 0 | 0 | 1 | 1 | 0 | 0 | 1 | 0 | 0 | 0 | 0 | 6 |
| Li et al., 2023 [18] | 1 | 0 | 0 | 0 | 0 | 0 | 1 | 0 | 0 | 0 | 0 | 0 | 1 | 0 | 0 | 0 | 1 | 0 | 0 | 0 | 0 | 4 |
| Li et al., 2024 [21] | 1 | 0 | 0 | 0 | 0 | 0 | 1 | 0 | 0 | 0 | 0 | 0 | 1 | 1 | 0 | 1 | 1 | 0 | 0 | 0 | 0 | 6 |

Note: A=Is the metric of time used in the statistical model reported, B=Is information presented about the mean and variance of time within a wave, C1=Is the missing data mechanism reported, C2=Is a description provided of what variables are related to attrition/missing data, C3=Is a description provided of how missing data in the analyses were dealt with, D=Is information about the distribution of the observed variables included, E=Is the software mentioned, F1=Are alternative specifications of within-class heterogeneity considered and clearly documented? If not, was sufficient justification provided as to eliminate certain specifications from consideration, F2=Are alternative specifications of the between-class differences in variance or covariance matrix structure considered and clearly documented? If not, was sufficient justification provided as to eliminate certain specifications from consideration, G=Are alternative shape/functional forms of the trajectories described, H=If covariates have been used, can analyses still be replicated, I=Is information reported about the number of random start values and final iterations included, J=Are the model comparison (and selection) tools described from a statistical perspective, K=Are the total number of fitted models reported, including a one-class solution, L=Are the number of cases per class reported for each model (absolute sample size, or proportion), M=If classification of cases in a trajectory is the goal, is entropy reported, N1=is a plot included with the estimated mean trajectories of the final solution, N2=Are lots included with the estimated mean trajectories for each model, N3=Is a plot included of the combination of estimated means of the final model and the observed individual trajectories split out for each latent class, O=Are characteristics of the final class solution numerically described, P=Are the syntax files available. The total score is the sum of all items.

**Supplementary Table S5 Associated factors and health-related outcomes of sleep health trajectories**

| Trajectory types | Sleep health dimensions |  | Associated factors | | | |  | Health-related outcomes | |
| --- | --- | --- | --- | --- | --- | --- | --- | --- | --- |
|  |  |  | Demographic | Socioeconomic | Physical | Psychological |  | Maternal | Infant |
| Single sleep trajectory | | | | | | | | | |
|  | Sleep duration |  |  |  |  | anxiety symptoms^[38]^ |  |  |  |
| Heterogeneous sleep trajectories | | | | | | | | | |
|  | Sleep quality |  | planned pregnancy^[16]^ | education^[15, 16, 20]^, income^[15]^, occupational stress^[33]^, employment^[17]^, social support^[14, 15, 33]^ | sleep quality^[15, 16, 35]^, fatigue symptoms^[14, 34]^, pre-pregnancy BMI^[34]^ | anxiety symptoms^[15, 35]^, depressive symptoms^[15, 16, 33-35]^ |  | PPD symptoms^[15, 16, 34]^, excessive body weight gain^[20, 34]^, postpartum fatigue symptoms^[34]^, postpartum mood disturbances^[35]^ |  |
|  | Sleep duration |  | age^[18]^, parity^[18]^ | education^[18, 20]^, employment^[18, 19]^ | health status^[18]^, pre-pregnancy BMI^[20, 21, 34]^ |  |  | PPD symptoms^[19]^, assisted delivery^[19]^, C-section^[19]^, preterm birth^[19]^ | overall development delay^[18]^, low birth length^[21]^, low birth weight^[19]^ |
|  | Sleep efficiency |  | race^[20]^ | education^[20]^ |  |  |  | C-section^[21]^, preterm birth^[21]^ | low birth weight^[21]^, low birth length^[21]^ |
|  | Sleep timing |  |  | education^[21]^ |  |  |  | preterm birth^[21]^ | low birth length^[21]^ |
|  | Insomnia symptoms |  | race^[22]^ |  |  | depressive symptoms^[22]^ |  | PPD symptoms^[22]^ |  |

Note: PPD = Postpartum symptoms, BMI = Body mass index, C-section = Caesarean section. Horwitz et al., 2023, Paul et al., 2019, Nagi et al., 2024 and Verma et al., did not report associated factors and health-related outcomes. [38] = van der Zwan et al., 2017, [16] = Bao et al., 2022, [18] = Li et al., 2023, [21] = Li et al., 2024, [14] = Lin-Lewry et al.,2023, [17] = Lyu et al., 2020, [34] = Tzeng et al., 2015, [35] = Wang et al., 2018, [33] = Zhang et al., 2023, [1-20] = Whitaker et al., 2021, [14-15] = Tomfohr et al., 2021, [21-22] = Sedov et al., 2020, [18-19] = Plancoulaine et al., 2017.

**Supplementary Figure S1 Heterogenous trajectories of sleep quality (n = 8)**

**
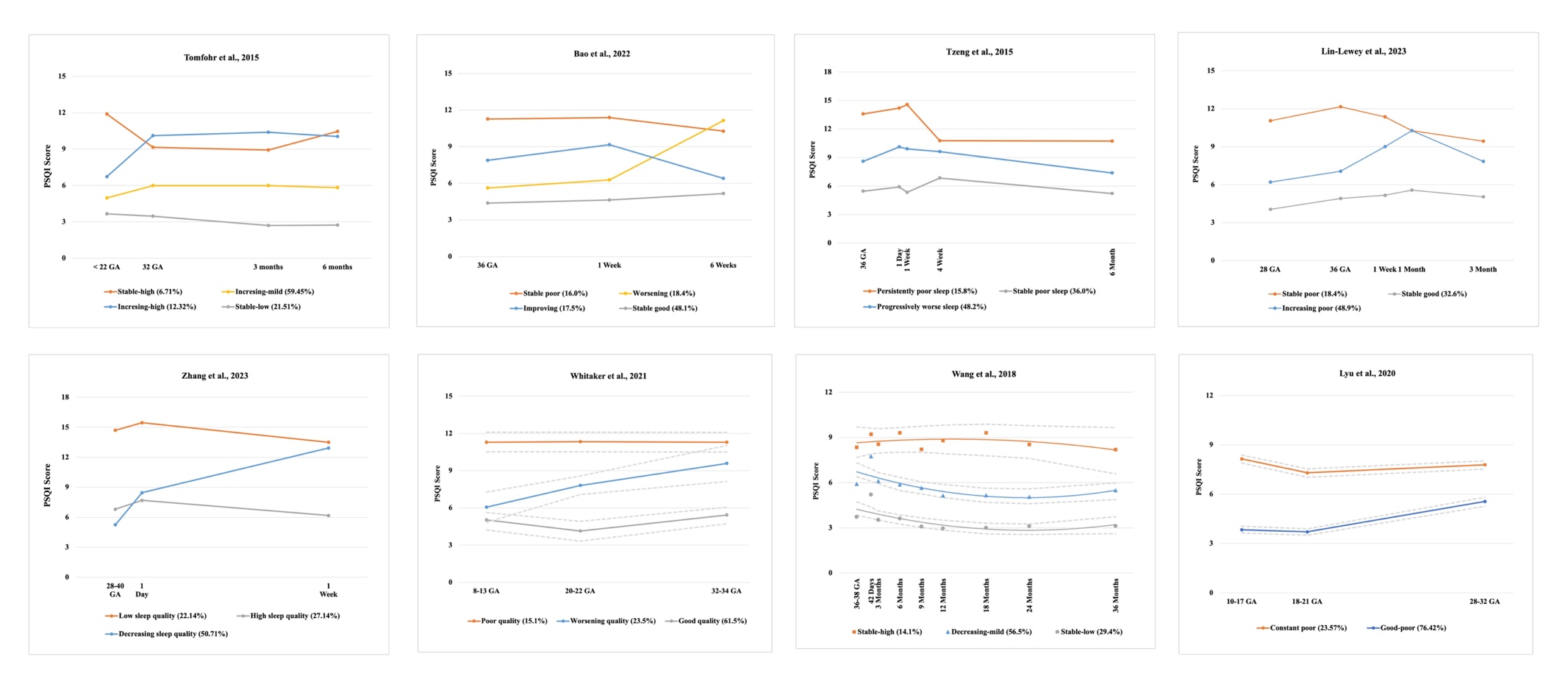
**

**Supplementary Figure S2 Heterogenous trajectories of sleep duration (n = 4)**

**
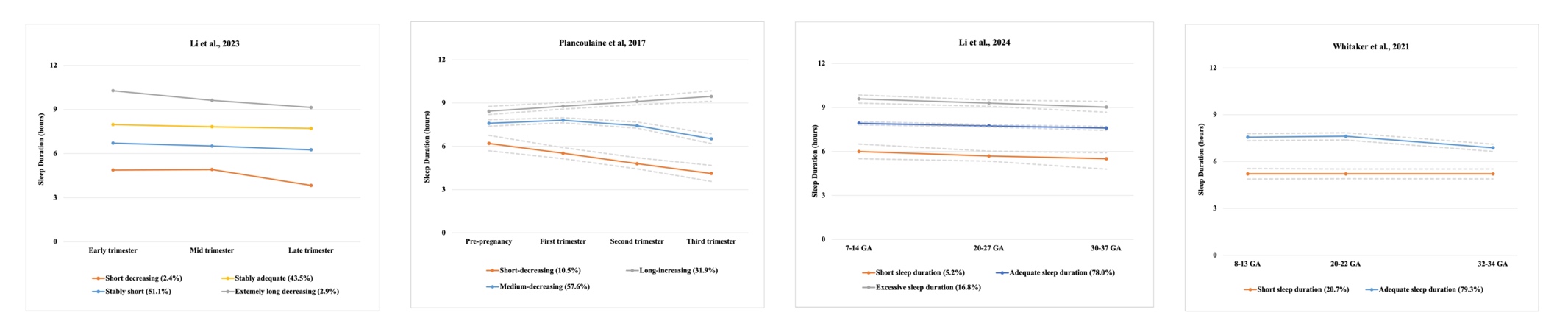
**

**Supplementary Figure S3 Heterogenous trajectories of sleep efficiency (n = 2)**

**
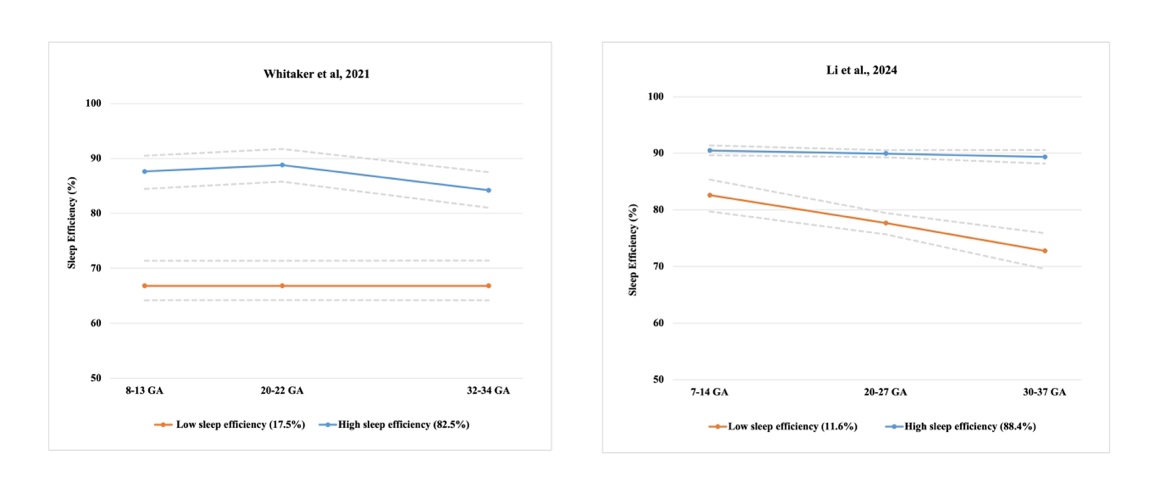
**

**Supplementary Figure S4 Heterogenous trajectories of sleep timing and insomnia symptoms (n = 2)**

**
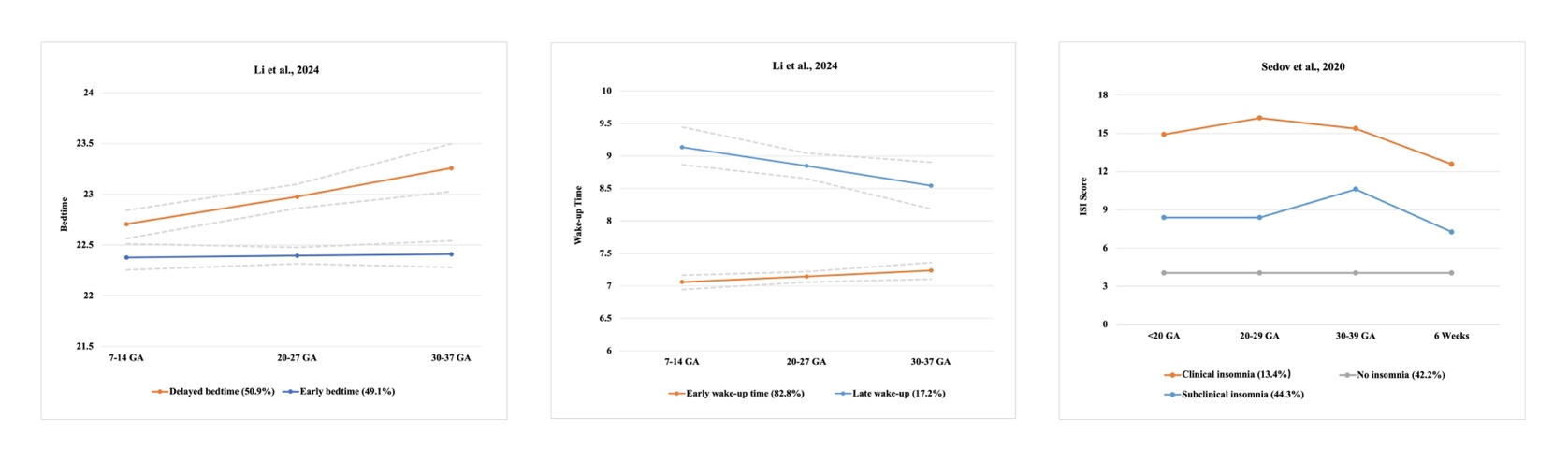
**

**Supplementary Figure S5 Meta-analysis of the prevalence of poor sleep quality trajectories** **(PSQI > 5) (n = 6)**

**
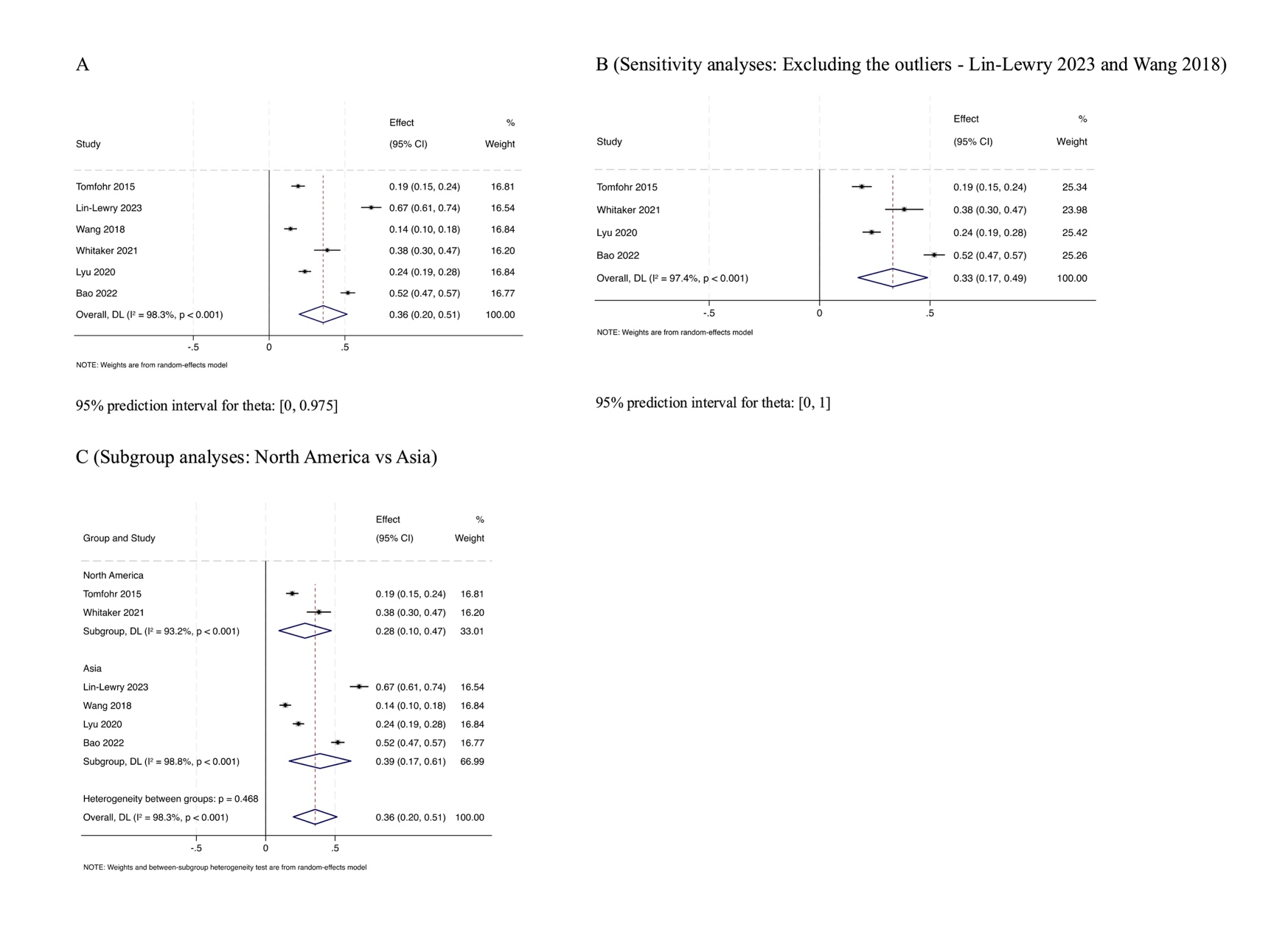
**

**Supplementary Figure S6 Meta-analysis of the prevalence of insufficient (< 6h) or excessive (> 8h) sleep duration trajectories (n = 4)**


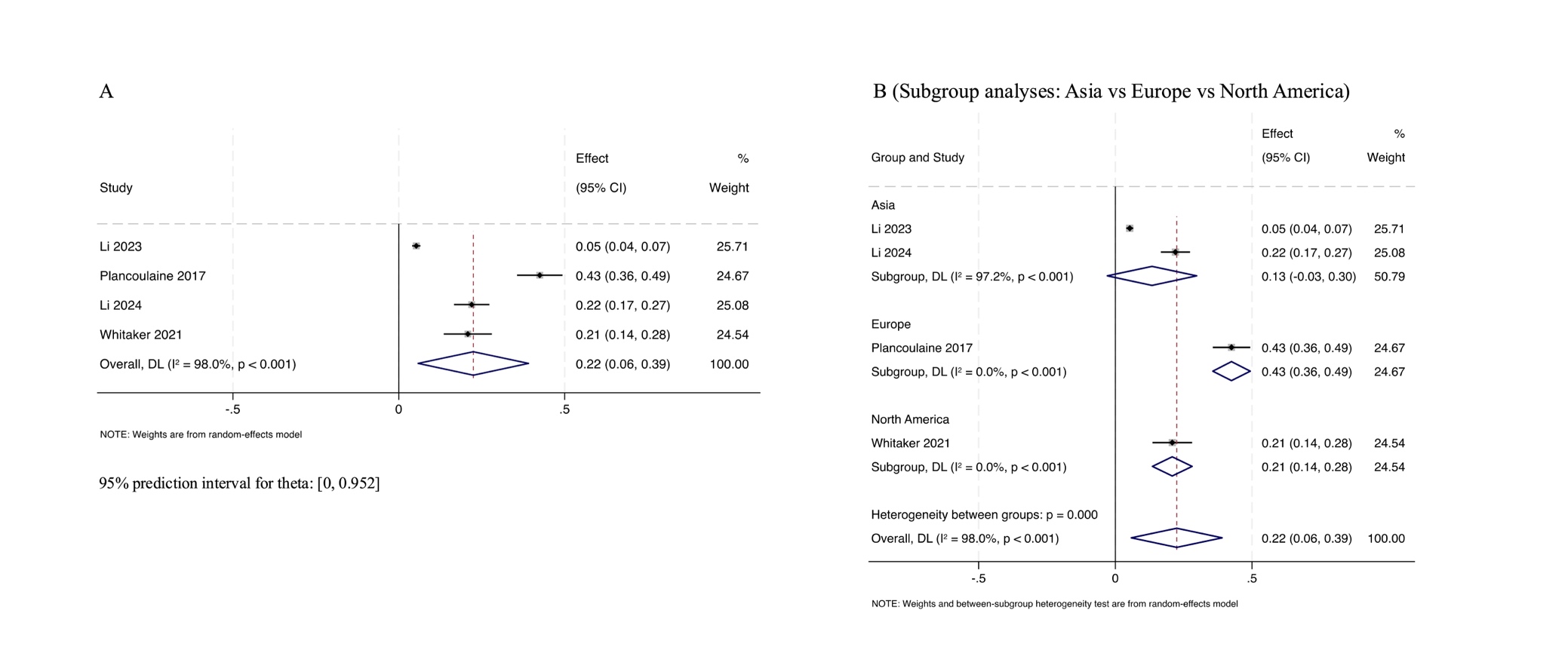

Supplement: zsaf095_suppl_Supplementary_Tables_S1-S5_Figures_S1-S6 [file zsaf095_suppl_supplementary_tables_s1-s5_figures_s1-s6.docx]
